# Evaluation of machine learning models for early prediction of gestational diabetes using retrospective electronic health records from current and previous pregnancies

**DOI:** 10.1136/bmjdhai-2025-000089

**Published:** 2025-12-18

**Authors:** Mark Germaine, Amy C O’Higgins, Brendan Egan, Graham Healy

**Affiliations:** 1 School of Computing, Dublin City University, Dublin, Ireland; 2 School of Health and Human Performance, Dublin City University, Dublin, Ireland; 3 Research Ireland Centre for Research Training in Machine Learning, Dublin City University, Dublin, Ireland; 4 UCD Centre for Human Reproduction, The Coombe Hospital, Dublin, Ireland; 5 Florida Institute for Human and Machine Cognition, Pensacola, Florida, USA

**Keywords:** Medical Records, Artificial intelligence, Decision Support Systems, Clinical, Electronic Health Records, Machine Learning

## Abstract

**Objective:**

To assess the performance of machine learning (ML) models in predicting gestational diabetes mellitus (GDM) using electronic health record (EHR) data from the first antenatal visit, and determine whether incorporating previous pregnancies data improves performance.

**Methods and analysis:**

In this retrospective cohort study, ML models were developed to predict GDM using EHR data (n=27 561, GDM 11.6%). Four ML algorithms: Logistic Regression (LR), Random Forest (RF), XGBoost (XGB) and Explainable Boosting Machine (EBM) were trained with seven to nine top clinical predictors from the EHRs. Models were trained and evaluated in separate first-trimester (n=27 561), nulliparous (n=11 623) and multiparous and past-pregnancy (n=4005) cohorts. Discrimination was measured by the area under the receiver-operating characteristic curve (AUC, 95 % CI), and calibration was assessed by slope and intercept. Decision-curve analysis was performed for models.

**Results:**

First-trimester models achieved AUC 0.819 (95% CI 0.811-0.827, slope=1.010, intercept=0.013) with LR, similar to more complex models such as XGB (AUC 0.818, slope=1.004, intercept=0.007), EBM (AUC 0.817, slope=0.988, intercept=-0.017) and RF (AUC 0.817, slope=1.062, intercept=0.103). Among nulliparous women, there was little difference between LR (AUC 0.813) and XGB, EBM or RF (0.805–0.814). When past pregnancy features were added to first-trimester data in multiparous women, discrimination improved: EBM AUC 0.885 (95% CI 0.867 to 0.900; slope 0.994), RF 0.878, LR 0.874 and XGB 0.876. Models using data from past pregnancies only achieved good discrimination (AUC 0.860, 95% CI 0.839 to 0.879; slope=1.028).

**Conclusion:**

A small panel of clinically selected variables provides robust early-pregnancy GDM prediction (AUC ∼0.81) and even stronger performance (AUC ∼0.86–0.89) when past pregnancy information is incorporated in multiparous women. Past pregnancy data alone gives useful preconception risk estimates. These findings highlight the promise of early GDM risk identification in both nulliparous and multiparous populations; however, additional research, including external validation and clinical trials, is needed to determine the models’ practical utility and effect on maternal and neonatal outcomes.

**WHAT IS ALREADY KNOWN ON THIS TOPIC**

- Machine learning (ML) approaches have been used to identify gestational diabetes mellitus (GDM) risk in early pregnancy, but most studies focus only on data from the current pregnancy and do not leverage prior obstetric history.

**WHAT THIS STUDY ADDS**

- Incorporating data from previous pregnancies substantially improved ML models’ predictive performance, and using past pregnancy data alone still yielded good performance, assessed by model discrimination, calibration and net benefit analysis, suggesting feasibility for real-time clinical use at or even before the first antenatal visit.

**HOW THIS STUDY MIGHT AFFECT RESEARCH, PRACTICE OR POLICY**

- These findings support the possibility of earlier GDM detection and intervention, and highlight the need for external validation and prospective trials to confirm broader utility, inform clinical workflows and guide policy on integrating ML-driven risk prediction into routine maternity care.

## Introduction

Gestational diabetes mellitus (GDM) is a form of diabetes presenting during pregnancy.^1^ A 2018 Lancet series^2–4^ underscored that lifestyle modifications initiated early in pregnancy could influence maternal and neonatal outcomes. However, the efficacy of such lifestyle interventions remains unclear, with several strategies offering limited benefits in terms of dietary^5 6^ and physical activity interventions,^7–9^ possibly due to the intervention’s commencement time, which is often during late stage pregnancy. Two meta-analyses^10 11^ on lifestyle interventions during pregnancy highlight this point: the timing of intervention is key. Lifestyle changes initiated during the first trimester were most effective in reducing GDM risk and improving maternal health. Furthermore, early interventions are expected to provide significant cost-saving benefits for healthcare systems by reducing complications associated with GDM.^12^

Machine learning (ML) offers a promising solution for the early identification of women at risk of GDM,^13^ addressing a key challenge of late stage (week 24–28) diagnosis. By leveraging large data sets, such as electronic health records (EHRs), ML creates predictive models that can identify women at increased risk for GDM before the traditional screening window.^14^ By identifying high-risk individuals at antenatal visits, or preconception,^15^ ML models could facilitate earlier lifestyle and clinical interventions. Such an approach could optimise the timing of interventions, potentially enhancing delivery outcomes and improving the health of both mother and child.^16 17^

Despite the growing evidence on ML models for GDM prediction,^18^ most studies using EHRs to date have been conducted in Asian populations,^19^ often yielding promising but population-specific results. Furthermore, most have primarily used data from the current pregnancy, with limited incorporation of past pregnancy information. One study incorporated data from a previous pregnancy to predict GDM, but it involved only East Asian women with prior GDM (n=553) and also used a current first-trimester glucose measurement.^20^ These constraints underscore the need for broader examinations of whether including previous pregnancy data can enhance early GDM prediction across diverse populations, including those in Europe, and whether such models can generalise beyond women already known to be at high risk.

Therefore, the aim of this study was to develop and evaluate the performance of ML models in predicting GDM using EHR data collected in the first trimester and to determine whether incorporating data from previous pregnancies could improve predictive performance. By including data from past pregnancies, the aim was to evaluate the potential for predicting GDM risk even before conception, thereby allowing for preconception risk assessment. Additionally, by developing models tailored to both nulliparous and multiparous populations, the study explored potential differences in performance while keeping in mind future applications in clinical decision support systems (CDSS).^21^ Finally, this study also investigated using a reduced set of clinically relevant features, recognising that limiting the feature set (variables) to be collected could enhance the practicality of model deployment by clinicians in a healthcare facility.

## Methods and analysis

### Study design and population

This retrospective cohort study employed ML techniques on EHRs from The Coombe Hospital, Dublin, spanning a 5-year period (2018–2022). The primary aim was to develop ML models that could predict the diagnosis of GDM by using EHR data collected during the first antenatal visit (∼12th week of gestation). Three distinct cohorts were used to build the ML models (see Study populations). The study was designed following the Transparent Reporting of a multivariable prediction model for Individual Prognosis Or Diagnosis (TRIPOD) +AI guidelines for reporting clinical prediction models.^22^ Patients or members of the public were not involved in the design, conduct, reporting or dissemination plans of this study due to its retrospective nature.

### Inclusion and exclusion criteria

Eligible records were from women who were 18 years or older, attended an antenatal visit at or before 16 weeks, with complete EHR data up to 16 weeks and validated GDM status. Exclusions included women with pre-existing diabetes (type 1 or 2), first antenatal visit after 16 weeks, those with missing or incomplete data for critical variables, invalidated GDM status and pregnancies from 2020 (see below).

### Study populations

Three distinct study populations were defined. The first-trimester population included all eligible pregnancies with data up to the 16th week of gestation. The nulliparous population, a subset of the general population, focused on women experiencing their first pregnancy. Finally, the multiparous population identified women with at least one previous pregnancy in the data set, allowing for the inclusion of historical data to predict GDM in subsequent pregnancies and modelling based on past pregnancy alone.

### Data source and validation

Data were routinely collected by trained midwives using standardised questionnaires and entered into the hospital’s electronic health system, Euroking K2 (Euroking Maternity Software Systems, UK).^23^ GDM was diagnosed according to the International Association of Diabetes and Pregnancy Study Groups (IADPSG) criteria.^1^ EHR entries indicating ‘Diabetes developed during pregnancy’ were labelled as GDM, while all others were labelled non-GDM. GDM status was cross-referenced with a real-time clinical database (2018–2022).^24^ Records were included only if both databases provided consistent labels. Data from 2020 were excluded due to COVID-19-related fluctuations in GDM detection.^24 25^ The final data set included 27 561 pregnancies and 108 retained variables. This sample size was deemed sufficient based on the *BMJ* guidelines for calculating the required sample size for developing a clinical prediction model.^26^ Individual prediction uncertainty for the clinical models was further assessed by calculating effective sample size.^27^

### Data cleaning and preprocessing

Data quality was ensured through extensive cleaning and preprocessing. Critical variables included maternal age, body mass index (BMI) at booking, key medical history items (eg, family history of diabetes, endocrine problems) and obstetric history (eg, previous GDM, parity). Pregnancy records missing any critical variables were excluded, and features with more than 20% missing data were removed unless the missing value could be interpreted as a null finding. Inconsistencies (eg, negative BMI) were either corrected if plausible or excluded if not. Categorical variables were simplified; for instance, the ‘Cardiac Problems’ feature, initially containing over 2600 string values, was simplified into a binary ‘NO’/‘YES’, with nulls interpreted as ‘NO’ after clinician consultation. Any remaining missing data were then removed.

Two deviations from this general approach were the ‘Occupation of the Mother’ mapped to the International Standard Classification of Occupations (ISCO)^28^ and grouped into skill level categories, with 4 representing the highest skill level and 0 representing unemployment. Second, birth weight percentiles were calculated using the weight of the baby and the gestational age at delivery, based on the percentile ranges presented by Nicolaides *et al*.^29^

For sequential pregnancies, two additional features, interpregnancy interval (days from the delivery date of one pregnancy to the conception date of the next) and interpregnancy weight gain (difference in maternal booking weight between successive pregnancies), were calculated.

### Model development

Four ML models were developed to address clinically relevant different aspects of the data: (1) first-trimester models with all data up to week 16 of gestation (routinely collected 11–13 weeks’ gestation), (2) nulliparous pregnancy models using first trimester data, (3) multiparous pregnancy models that incorporated previous and interpregnancy features to predict GDM and (4) past pregnancy models that use data only available from the previous pregnancy to predict future GDM. Model cohort information and the predictors used are presented in table 1 and a list of all variables in the EHR is reported in the online supplemental table 5. The chosen ML algorithms were Random Forest Classifier (RF), Logistic Regression (LR), XGBoost Classifier (XGB) and Explainable Boosting Machine (EBM). LR has historically been popular in GDM modelling,^18^ but recent evidence suggests that more advanced models like RF and XGB yield better results.^30 31^ EBMs have been shown to match the performance of these advanced models while retaining interpretability.^32^ A Dummy Classifier from scikit-learn was included as a baseline to establish a minimal threshold for performance.

10.1136/bmjdhai-2025-000089.supp3 Supplementary data

**Table 1:** Model data sets

Model data set	No. pregnancies	Predictors	GDM %
First-trimester population	27 561	n=9 Ethnicity, family history of diabetes (binary), previous history of GDM (binary), endocrine problems (PCOS/thyroid conditions, binary), parity (integer), age (integer), BMI (float), blood pressure (float).	11.6
Nulliparous population	11 623	n=7 Ethnicity, family history of diabetes (binary), endocrine problems (PCOS/thyroid conditions, binary), age (integer), BMI (float), blood pressure (float).	9.9
Multiparous population	4005	n=8 Ethnicity, age (integer), interpregnancy weight change (float), history of GDM (binary), BMI (float), time between pregnancies (integer), birth weight percentile (float), family history of diabetes (binary).	12.2
Past pregnancy	4005	n=6 Ethnicity, age (integer), history of GDM (binary), BMI (integer), birth weight percentile (float), family history of diabetes (binary).	12.2

BMI, body mass index; GDM, gestational diabetes mellitus; PCOS, polycystic ovary syndrome.

### Data preprocessing

Clinically relevant features were selected by modelling the full EHR, retaining the 15 highest-importance variables (according to SHapley Additive exPlanations (SHAP) plots), and applying backward elimination until cross-validated performance, measured by area under the receiver operating characteristic (ROC) curve (AUC), fell; we also dropped ISCO skill level given its limited availability to maximise external validity. A pipeline was developed for data preprocessing and modelling. Categorical variables underwent one-hot encoding with the first category dropped, and numerical variables were standardised using StandardScaler. Scikit-learn’s ColumnTransformer ensured separate treatment of categorical and numerical data, and the processed DataFrame retained patient identifiers to facilitate group-based splitting. To avoid data leakage, the data set was split into training and test sets using GroupShuffleSplit, ensuring all records from the same patient were allocated to the same split. This approach ensured patient-specific splits, providing separate data for training, hyperparameter tuning and model evaluation.

### Model training

Hyperparameter tuning using RandomizedSearchCV with a stratified k-fold cross-validation (CV) strategy was used for each model. This approach involved a randomised search over specified parameter ranges (see online supplemental table 1) with 10 iterations, evaluating model performance on multiple splits of the training set. External validation was attempted but we were unable to source an external data set.^19^ The final hyperparameters for each model are reported in online supplemental table 4.

### Model performance evaluation

The performance of the ML models was primarily evaluated using fivefold CV to generate out-of-fold predictions, with the AUC used to measure discrimination. Bootstrapping (1000 iterations) was employed to compute 95% CIs for the AUC, each created by sampling, with replacement, the same number of observations as the full data set.^33^ Model calibration was assessed both qualitatively with the use of calibration plots and quantitatively, with the use of slope, intercept and observed to expected ratio (O:E ratio), as has been recommended for clinical prediction models.^34^ AUC measures the model’s ability to distinguish between classes without being sensitive to class imbalances,^35^ while calibration assesses the agreement between the observed and predicted outcomes. Models were further evaluated for net clinical benefit with decision-curve analyses.^36 37^ Model performance was further assessed using the Average Precision (AP) Score, confusion matrices (online supplemental figure 1), model sensitivity, specificity and F-1 score with a default threshold of 0.5 for determining the binary classification from the classifier output, while Brier score was calculated to measure agreement between predicted probabilities and observed outcomes across all models.^38^ Final performance was obtained with a fivefold stratified outer CV on the entire data set: each hyperparameter tuned model was fitted on 4/5 of the data and used to predict the held-out 1/5, producing out-of-fold predictions for every pregnancy. From these out-of-fold predictions, we computed: AUC (95% bootstrap CI), AP, sensitivity, specificity, F1, Brier score, calibration slope/intercept and O:E ratio and aggregated ROC/precision-recall (PR) curves.

10.1136/bmjdhai-2025-000089.supp1 Supplementary data

Further, to determine whether the models’ predictive capabilities generalised across diverse patient backgrounds, performance metrics were also stratified by ethnicity. The AUCs were analysed with a linear mixed-effects model in which ethnicity was a fixed factor and each data set×classifier row was entered as a random intercept, thereby treating the six ethnicity-specific AUCs produced by the same model as repeated measures. Post-hoc Sidak-adjusted contrasts tested every ethnicity against the Caucasian reference group.

### Addressing class imbalance

The data set exhibited class imbalance, with a GDM rate of 11.6%. Pilot experiments with the Synthetic Minority Over-sampling Technique did not yield improvements, and adjusting class weights resulted in a bias toward higher false positives compared with false negatives. This trade-off was considered undesirable for clinical application. Therefore, no further techniques were applied.

### Feature importance and interpretability

Feature importance was determined after each model had been trained on 80% of the data (training set) with the remaining 20% reserved as a test set, ensuring that no information from the test set influenced model parameters. Relative importance scores from RF (feature_importances_) and coefficient-derived ORs from LR provided basic importance measures. In XGB, SHAP summarised each feature’s contribution to predictions, while EBM’s built-in interpretability tools offered global and per-feature visualisations (interpret package). Although SHAP values were computed on the test data for final interpretability assessments, all feature importance metrics reflected the model parameters learnt from the training set only.

### Software and computational resources

Model development was conducted in Python V.3.8.8, with pandas (V.1.2.4) for data manipulation, numpy (V.1.23.5), scikit-learn (V.1.2.1) for ML workflows, seaborn (V.0.11.1) and matplotlib (V.3.3.4) for visualisation, xgboost (V.1.7.6), shap (V.0.41.0) and interpretML (V.0.3.0). Computations were carried out on an Apple M1 workstation with 16 GB RAM. Training times ranged from several minutes for models (eg, LR) to longer durations for EBM.

## Results

### Population characteristics

Initially, 37 651 pregnancy records were identified, of which 10 090 were excluded (2696 for missing/incomplete data and 7394 from 2020), resulting in N=27 561 for analysis. Table 2 presents the baseline characteristics of the study participants across these populations, including key demographics, medical history and clinical measurements. Further details of baseline characteristics by ethnicity are provided in the online supplemental table 2.

**Table 2:** Patient characteristics of the validated data set

Characteristics	N=27 561 (%)	Rate of GDM (%)
Ethnicity		
Caucasian	24 180 (87.8)	10.0
Black African	554 (2.0)	14.3
Southeast Asian	1360 (4.9)	33.9
Other	824 (3.0)	13.7
Asian	489 (1.8)	21.7
Middle Eastern	154 (0.6)	13.6
Family history of diabetes		
No	21 154 (76.7)	8.4
Yes	6407 (23.3)	21.9
Previous history of GDM		
No	26 483 (96.1)	9.5
Yes	1078 (3.9)	62.5
Other endocrine problems		
No	21 707 (78.8)	8.5
Yes	5854 (21.2)	22.8
Current GDM		
No	24 373 (88.4)	0
Yes	3188 (11.6)	100
Parity		
0	11 377 (41.3)	9.9
1	9875 (35.8)	11.4
≥2	6309 (22.9)	15.0
Age		
Age (mean±SD)	32±5	
≥40	2178 (7.9)	17.2
<40	25 383 (92.1)	11.1
BMI		
BMI (mean±SD)	26.2±5.3	
<25	13 695 (49.7)	4.9
25 to <30	8423 (30.6)	11.6
30 to <35	3537 (12.8)	24.2
35 to <40	1280 (4.6)	33.0
≥40	626 (2.3)	41.9
BP		
Systolic BP (mean±SD)	111±11	
Diastolic BP (mean±SD)	67±8	

BMI, body mass index; BP, blood pressure; GDM, gestational diabetes mellitus.

### Discrimination and calibration of GDM prediction models

With nine routinely recorded predictors, the first-trimester LR model attained an AUC of 0.819 (95% CI 0.811 to 0.827), calibration slope 1.010, intercept 0.013 and AP 0.441. RF, XGB and EBM showed virtually identical discrimination (AUC 0.817–0.818) and good calibration (slopes 0.988–1.062, intercepts −0.017 to 0.103).

Among the nulliparous pregnancies, EBM achieved the highest AUC (0.814, 95% CI 0.799 to 0.827), with slope 1.004, intercept 0.017 and AP 0.342; LR, XGB and RF ranged from 0.805 to 0.813. Brier scores were 0.076–0.077 and specificity exceeded 0.98 for all models, although sensitivity remained modest (0.05–0.11).

In multiparous women, using previous-pregnancy variables resulted in higher discrimination. EBM reached an AUC of 0.885 (95% CI 0.867 to 0.900), slope 0.994 and intercept 0.000, with an AP of 0.620, sensitivity 0.464 and Brier score 0.068. RF, LR and XGB followed closely (AUC 0.874–0.878). Calibration intercepts stayed within ±0.10 and O:E ratios approximated 1.0.

Using past pregnancy features alone, EBM maintained good performance (AUC 0.860, 95% CI 0.839 to 0.879; slope 1.028; intercept 0.051; AP 0.556) and RF, LR and XGB yielded AUC values between 0.854 and 0.858. Probability calibration remained acceptable (slopes 0.935–1.028, intercepts −0.094 to 0.051).

**Figure 1:**
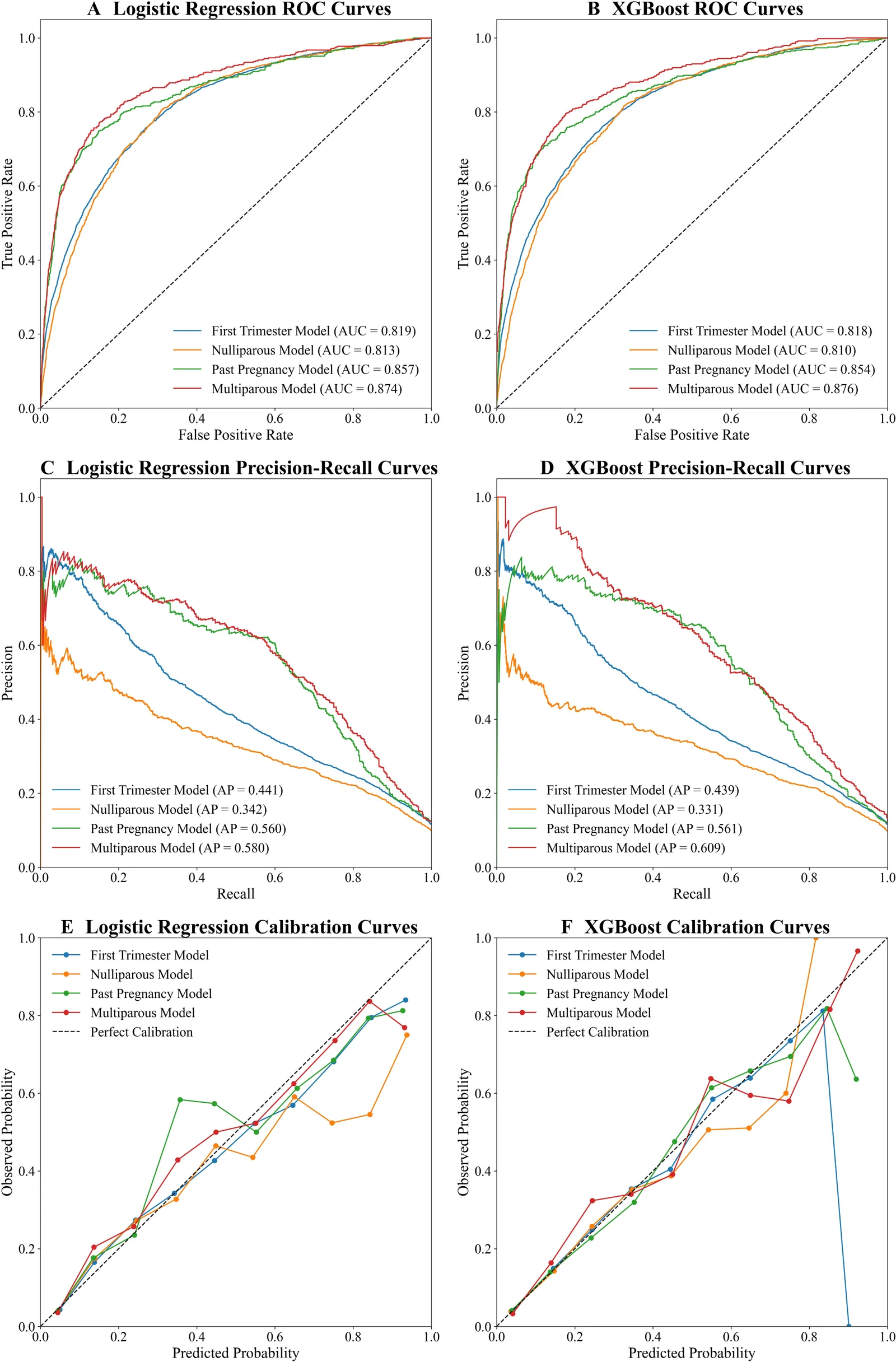
Model performance and calibration for Logistic Regression (left panel) and XGBoost (right panel). Subplots A, C and E show the AUC, AP and calibration plots, respectively, for the Logistic Regression models, while subplots B, D and F show the corresponding plots for the XGBoost models. AP, average precision; AUC, area under the receiver-operating characteristic curve; ROC, receiver-operating characteristic.

The full performance metrics are reported in table 3. Across all settings, Brier scores clustered between 0.068 and 0.083, indicating good calibration, with predicted probabilities closely aligning with observed outcomes. AUC curves for the LR and XGB models are shown in figure 1A,B, the corresponding PR curves in figure 1C,D and the calibration plots in figure 1E,F.

**Table 3:** Comparative performance metrics of machine learning models evaluated using out-of-fold predictions across various data sets for selected predictors

Data set and model	AUC (95% CI)	Calibration slope	Calibration intercept	O:E ratio	AP	Sensitivity	Specificity	F1 score	Brier score
First-trimester models									
Random Forest Classifier	0.817 (0.808 to 0.825)	1.062	0.103	1.001	0.439	0.184	0.989	0.291	0.082
Logistic Regression	0.819 (0.811 to 0.827)	1.010	0.013	0.997	0.441	0.217	0.984	0.324	0.082
XGBoost Classifier	0.818 (0.810 to 0.826)	1.004	0.007	1.000	0.439	0.193	0.988	0.301	0.082
Explainable Boosting Machine	0.817 (0.809 to 0.826)	0.988	−0.017	1.002	0.442	0.199	0.987	0.307	0.082
Dummy Classifier	0.497 (0.484 to 0.511)	−0.001	−1.993		0.122	0.107	0.887	0.111	0.209
Nulliparous models									
Random Forest Classifier	0.805 (0.791 to 0.819)	0.974	−0.042	1.002	0.319	0.053	0.995	0.095	0.077
Logistic Regression	0.813 (0.799 to 0.827)	0.995	−0.008	1.001	0.342	0.112	0.988	0.184	0.076
XGBoost Classifier	0.810 (0.795 to 0.823)	0.968	−0.054	1.001	0.331	0.066	0.993	0.117	0.076
Explainable Boosting Machine	0.814 (0.799 to 0.827)	1.004	0.017	1.008	0.342	0.085	0.992	0.147	0.076
Dummy Classifier	0.503 (0.474 to 0.535)	0.001	−2.196		0.098	0.108	0.899	0.106	0.178
Multiparous models									
Random Forest Classifier	0.878 (0.859 to 0.894)	1.073	0.099	0.997	0.618	0.431	0.978	0.543	0.069
Logistic Regression	0.874 (0.855 to 0.891)	1.030	0.048	1.001	0.580	0.441	0.970	0.533	0.071
XGBoost Classifier	0.876 (0.857 to 0.893)	1.031	0.012	0.980	0.609	0.423	0.976	0.529	0.070
Explainable Boosting Machine	0.885 (0.867 to 0.900)	0.994	0.000	1.005	0.620	0.464	0.974	0.620	0.068
Dummy Classifier	0.459 (0.429 to 0.492)	−0.013	−2.290		0.123	0.065	0.852	0.062	0.248
Past pregnancy models									
Random Forest Classifier	0.858 (0.837 to 0.875)	0.976	−0.037	0.998	0.549	0.447	0.973	0.544	0.072
Logistic Regression	0.857 (0.836 to 0.876)	0.995	−0.008	0.999	0.560	0.441	0.965	0.521	0.072
XGBoost Classifier	0.854 (0.832 to 0.873)	0.935	−0.094	1.002	0.561	0.454	0.972	0.548	0.072
Explainable Boosting Machine	0.860 (0.839 to 0.879)	1.028	0.051	1.004	0.556	0.443	0.970	0.533	0.072
Dummy Classifier	0.459 (0.429 to 0.492)	−0.013	−2.290		0.123	0.065	0.852	0.062	0.248

AP, Average Precision Score; AUC, area under the receiver operating characteristic curve; O:E ratio, observed to expected ratio.

### Net clinical benefit

**Figure 2:**
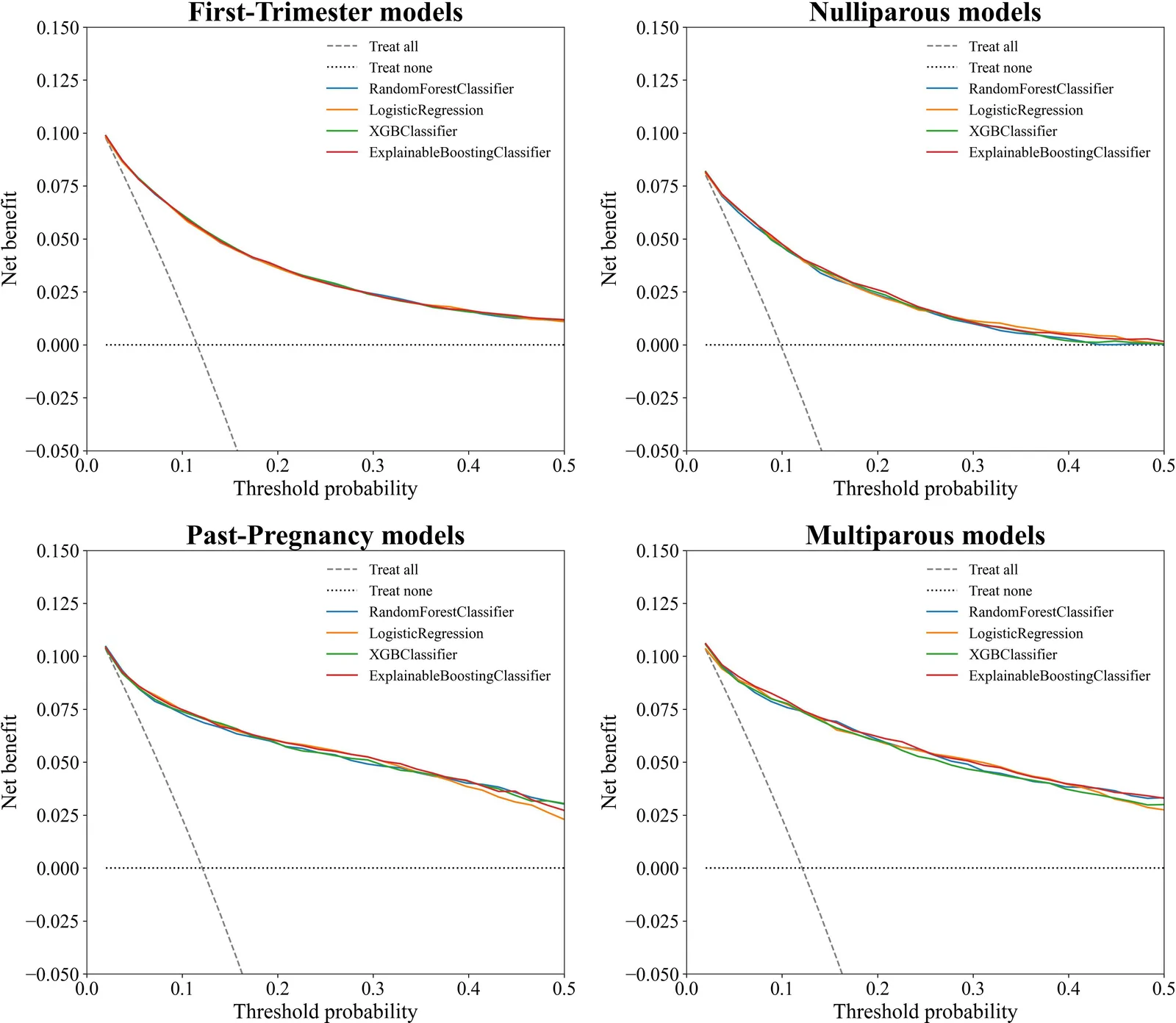
Decision-curve analysis for the four different model populations.

Decision-curve analysis (figure 2) was used to translate statistical performance into clinical utility.^34 36 37^ Net benefit peaks around the lower thresholds (∼0.10 at a threshold of 0.05) and tapers as the threshold rises, reflecting the usual trade-off between missed cases and false positives. Curves for RF, LR, XGB and EBM almost overlap, indicating negligible differences in clinical utility once discrimination and calibration are comparable. The multiparous models, boosted by previous-pregnancy information, achieve the highest net benefit, whereas the nulliparous curves approach the treat-none line sooner, mirroring their lower sensitivity. Crucially, none of the model curves fall below the treat-none baseline at thresholds <0.5, suggesting that use of these models would not introduce net clinical harm.

### Model performance across ethnicities

Performance metrics stratified by ethnicity are shown in online supplemental table 3. A linear mixed-effects model with a random intercept for each data set×classifier row found a significant effect of ethnicity on model AUC (*F*_(5, 99.5)_=88.36, p<0.001). Compared with Caucasian patients (mean AUC 0.84), discrimination was significantly lower in black African patients (AUC 0.772; p<0.001), Asian patients (AUC 0.757; p<0.001) and Southeast Asian patients (AUC 0.755; p<0.001), and significantly higher in the ‘Other’ category (AUC 0.903; p<0.001). Discrimination in Middle Eastern patients (AUC 0.828) did not differ significantly (p=0.78). Generally, the models maintained performance for Caucasian patients (AUC >0.8); however, there was reduced performance in some smaller or more diverse subgroups. Conversely, the models continued to perform well in the Other category, although small sample sizes may have skewed these results. For the FTP-9 model, no individuals in any ethnic group had n*_eff_* <30. For the SPP-8 model, a notable proportion of individuals in ethnic groups had n*_eff_* <30: 100% for Middle Eastern, 30% for black African and 25% for Asian.

### Feature importance

The feature importance analysis identified the top predictors of GDM, including a history of GDM, maternal BMI at booking, maternal age, family history of diabetes, ethnicity, interpregnancy weight gain and time between pregnancies. The SHAP plots for the XGB are shown in online supplemental figure 2. The feature importance for all of the models can be found in online supplemental figure 2.

10.1136/bmjdhai-2025-000089.supp2 Supplementary data

## Discussion

This study evaluated the performance of several ML models for early prediction of GDM using data available at the first antenatal visit and examined whether incorporating information from previous pregnancies enhances predictive performance. Overall, the findings confirmed that incorporating prior pregnancy history can improve early risk prediction for GDM. For example, the multiparous models that included both first-trimester and previous pregnancy features achieved an AUC of 0.885, higher than models using first-visit data alone, suggesting that a woman’s obstetric history is highly informative for forecasting GDM in a subsequent pregnancy. Notably, even a simplified model using only past pregnancy features achieved discrimination of AUC 0.860, highlighting that a focused set of clinical predictors from previous pregnancies can achieve most of the predictive signal.

In the first-trimester models, the LR model performed similarly to more complex models such as XGB and EBM (AUC ∼0.82). This contrasts with much of the existing literature, highlighted in a recent meta-analysis that found LR models achieved a pooled AUC of 0.815, compared with 0.889 for non-linear models.^18^ This discrepancy suggests that more sophisticated ML algorithms tend to perform better, potentially capturing complex, non-linear relationships in the data. However, the comparisons in the meta-analysis were not always direct comparisons of model performance, as the models were often tested on different data sets, each with varying characteristics, sample sizes and feature availability. Nevertheless, even when looking at studies that implemented LR alongside more advanced models on the same data set, the pattern of increased performance with non-linear models like XGB or RF remains evident.^39–43^ The lack of a gap in LR performance may indicate that the relationships between early-pregnancy predictors and GDM are largely linear or additive, meaning a well-specified linear model can capture them adequately. It is also possible that the complex models were limited by the data quality or volume, or by the fact that only routine, non-invasive features were intentionally used. This underscores an important point for clinical ML; more complex is not always better, especially if interpretability and ease of use are priorities. From a clinician’s perspective, a simpler model that offers similar performance might be preferable for integration into practice, due to its transparency and reliability.^44^

A strength of this study is the exclusive use of non-invasive, routinely collected data available at booking, which enhances the practicality and scalability of implementing the model in clinical settings. With the exception of height, body mass and blood pressure, all predictors in the models were questionnaire-based or existing records. Among previous studies that used non-invasive features, the majority demonstrate moderate predictive power (AUC 0.7–0.8),^13^ with two exceptions to date that demonstrate much better performance.^39 45^ In comparison, our models that used non-invasive features achieved AUCs ranging from 0.819 in the first-trimester models up to 0.885 in the multiparous models that included obstetric history, suggesting that leveraging a patient’s obstetric history and optimising the ML methodology can substantially boost performance even without biochemical markers. Notably, the multiparous EBM model demonstrated the highest performance, achieving an AUC of 0.885, which is in the range reported by Belsti *et al*^39^ (0.921) and Sweeting *et al*^45^ (0.880), and in the range of models that use biochemical predictors in addition to non-invasive features.^41^

Using only previous pregnancy variables in multiparous women improved model performance compared with the first-trimester and nulliparous models, with EBM achieving an AUC of 0.860. These results align favourably with findings from other studies that emphasise the importance of early pregnancy and preconception data for GDM prediction, particularly when considering non-invasive data collection. For instance, Artzi *et al*^46^ used features accessible ‘at the beginning of pregnancy’ and achieved an AUC of 0.799 using just nine features collected from questionnaires. It could be argued that this is a similar approach to the first-trimester models without blood pressure measures, which have minimal impact on the model. Additional work has demonstrated the benefit (AUC 0.930) of incorporating biochemical markers like glycosylated haemoglobin (HbA1c) with other features collected during the preconception stage of pregnancy.^15^ Thus, these data demonstrate the potential for predicting diagnosis of GDM with data available prior to conception. The inclusion of interpregnancy factors, such as weight gain and time interval between pregnancies, provided an additional perspective in our study, enhancing the models’ predictive power to an AUC of 0.885. This finding aligns with work demonstrating the value of including more comprehensive data in the prediction of recurrent GDM, which achieved an AUC of 0.942 with Light Gradient Boosting and 0.924 with XGB, by incorporating biomarkers such as the oral glucose tolerance test (OGTT) results from the index pregnancy, and fasting plasma glucose (FPG) and triglycerides (TG) in the first trimester of the ongoing pregnancy.^20^

The reported AUC values in our study demonstrate consistency across some subgroups, which is important for ensuring generalisability of the models, while performing poorly across others. The nulliparous models achieved AUC of ∼0.81, indicating promising predictive performance in identifying diagnosis of GDM among nulliparous pregnancies. While these figures appear higher than those reported in other studies that have focused on nulliparous pregnancies—such as Kang *et al*,^47^ who reported variability in AUC between multiparous (0.720) and nulliparous (0.672), and Cooray *et al*^48^ (AUC 0.732) and Donovan *et al*^49^ (AUC 0.710), both of which focused on nulliparous populations using LR—direct comparisons should be interpreted cautiously. The underlying cohorts differed in age, BMI, ethnicity, GDM prevalence and data-collection protocols, and our models have not yet been externally validated. Nonetheless, the consistency of performance within our own cohort suggests that the selected predictors capture risk factors for nulliparous women, warranting further validation in independent populations.

Beyond parity-based analysis, we also explored model performance across ethnic subgroups (see online supplemental table 3). While results remained consistent among Caucasian participants, performance declined in certain minority groups, particularly Asian, Southeast Asian and black African populations. This finding is consistent with previous research in Ireland demonstrating a marked decrease in model performance in non-Caucasian populations.^50^ In contrast, models continued to perform favourably for Other and Middle Eastern individuals, though the small sample sizes for these subgroups limit the generalisability of these findings. However, models trained on diverse populations in California, USA, noted the opposite trend, whereby models performed well in Hispanic populations but tended to underperform in Caucasian populations.^49^ This highlights the need for including more diverse data in model training.

The feature importance analysis confirmed that a history of GDM, maternal BMI, maternal age and ethnicity were strong predictors in the early pregnancy GDM models and aligned with meta-analytical analysis in this field.^18^ Other frequent predictors that were not available in the current feature set were FPG, TG and HbA1c. In our multiparous models, interpregnancy weight gain also emerged as a significant predictor, consistent with previous work.^20^ These findings are confirmatory, as they reinforce the known clinical relevance of these features and their effectiveness as strong predictors in different studies and populations.

In this study, we developed subset versions of each model by selecting the most clinically relevant features, aiming to enhance the potential for CDSS^21^ by relying on a limited set of predictors rather than an entire EHR. The models presented here represent similar model performance to training on the full EHRs (online supplemental table 6), with the reduced risk of overfitting to the data. This streamlined approach could facilitate earlier GDM risk prediction, potentially as early as 12 weeks’ gestation or even preconception, thereby enabling more proactive interventions such as earlier diagnostic testing long before standard screening. However, to integrate these models seamlessly into antenatal workflows, clinicians need clear guidelines for classifying high-risk and moderate-risk patients, and the chosen thresholds must carefully balance the risks of false positives (unnecessary interventions) and false negatives (missed high-risk cases).

This study has several limitations. First, the data set used for model development was derived from a single institution, which may limit the generalisability of the findings. External validation using data from other populations is necessary to confirm the models’ robustness, as emphasised by others,^42 49 51^ who validated their model performance across independent populations. The use of historical data from previous pregnancies also means that model performance may vary based on the quality and availability of such data across healthcare systems. The method by which features were pre-processed could have resulted in a loss of potentially important information. One-hot encoding broad categorical features, such as ‘Endocrine problems’, into binary variables may have discarded valuable context that could have improved predictions. Moreover, we did not have access to blood-based biomarkers in this study, such as FPG or HbA1c, which have been shown to be strong predictors of GDM.^15^ While the goal of this study was to develop a model that could be used at the booking visit in the first trimester without the need for extensive laboratory tests, incorporating point-of-care biomarkers could further enhance model performance. Socioeconomic and educational status were also not directly available in this data set. Previous research has shown that educational attainment, particularly in women, is correlated with health outcomes, including the risk of GDM and interpregnancy weight gain.^52^ The hospital in this study does not universally screen women for GDM, suggesting that some of the data set contains undiagnosed GDM,^53^ despite best efforts to validate the labels.^24^ Further, because SHAP, EBM plots and other tree tools define importance differently, using one method (eg, SHAP) across all models might slightly alter explanations but not our performance conclusions. Finally, our models did not include OGTT results, limiting predictions to the IADPSG criteria. Modelling GDM using OGTT results could provide a more versatile tool applicable to multiple diagnostic standards.

In conclusion, these ML models, particularly those incorporating data from previous pregnancies, have the potential early in pregnancy to identify women at greater risk of later diagnosis of GDM. Early identification may allow for timely interventions, which could mitigate the adverse maternal and foetal outcomes associated with GDM. Future research should focus on validating the developed models in external data sets to assess their generalisability. Incorporating additional features, such as lifestyle factors and biomarkers, may further improve model performance and sensitivity. Additionally, prospective studies evaluating the integration of these models into clinical workflows would be valuable to determine their impact on clinical decision-making and patient outcomes.

## Data Availability

No data are available. Due to patient confidentiality and data use agreements, individual-level data cannot be shared publicly. The models are intended for research purposes and require external validation before clinical implementation.
