# Supplementary material for: Evaluation of machine learning models for early prediction of gestational diabetes using retrospective electronic health records from current and previous pregnancies
Source: BMJ Digit Health Ai. 2025 Dec 18;1(1):e000089. doi: 10.1136/bmjdhai-2025-000089 (PMC13492532; doi:10.1136/bmjdhai-2025-000089)
Supplement: Supplementary file 1 [file bmjdhai-1-1-bmjdhai-2025-000089-s001.docx]

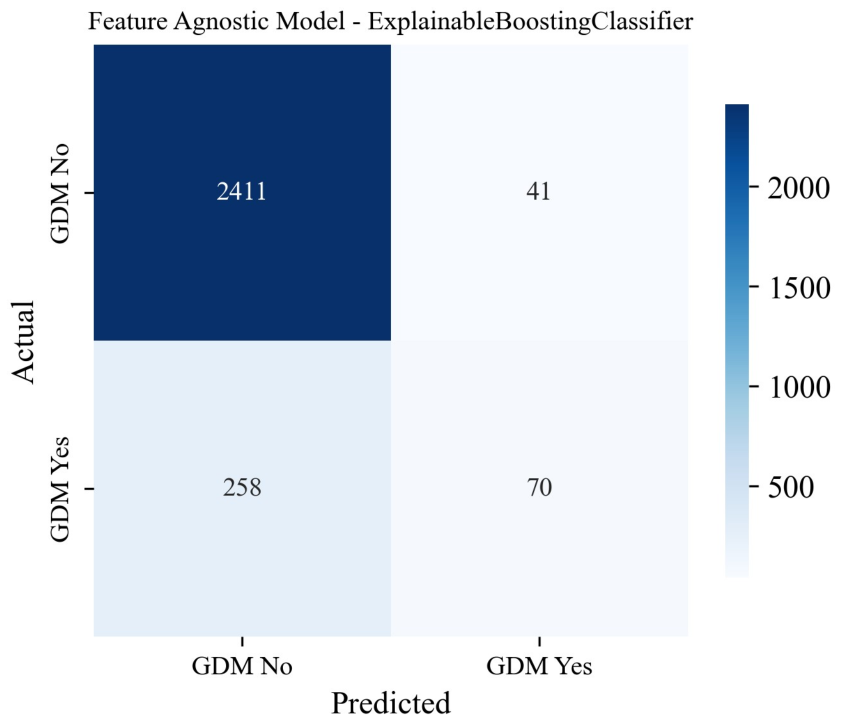
Feature Agnostic Model - ExplainableBoostingClassifier
Actual
GDM No
GDM Yes
2411
41
258
70
GDM No
GDM Yes
Predicted
2000
1500
1000
500

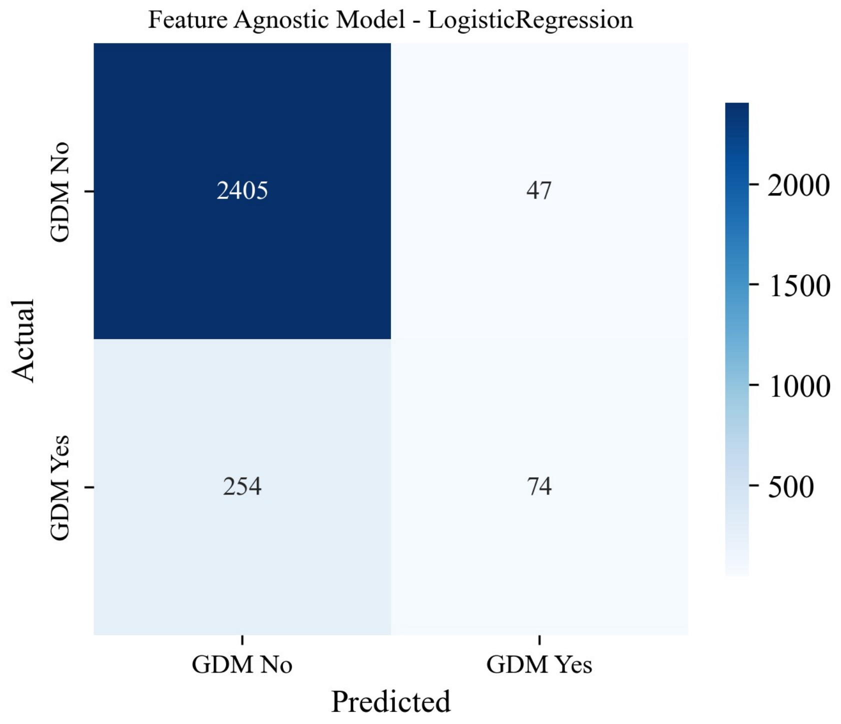
Feature Agnostic Model - LogisticRegression
Actual
GDM No
GDM Yes
2405
47
254
74
GDM No
GDM Yes
Predicted
2000
1500
1000
500

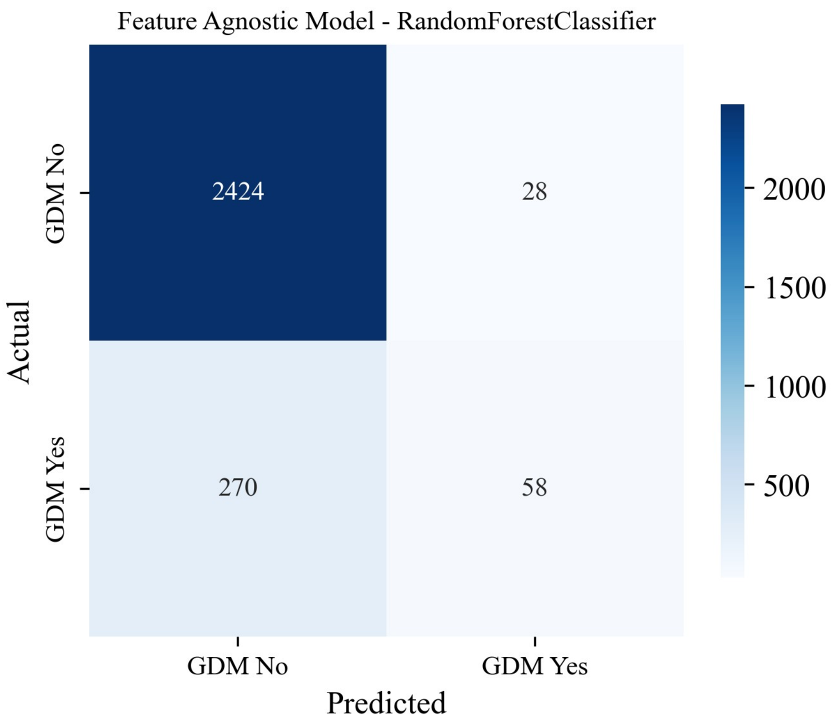
Feature Agnostic Model - RandomForestClassifier
Actual
GDM No
GDM Yes
2424
28
270
58
GDM No
GDM Yes
Predicted
2000
1500
1000
500

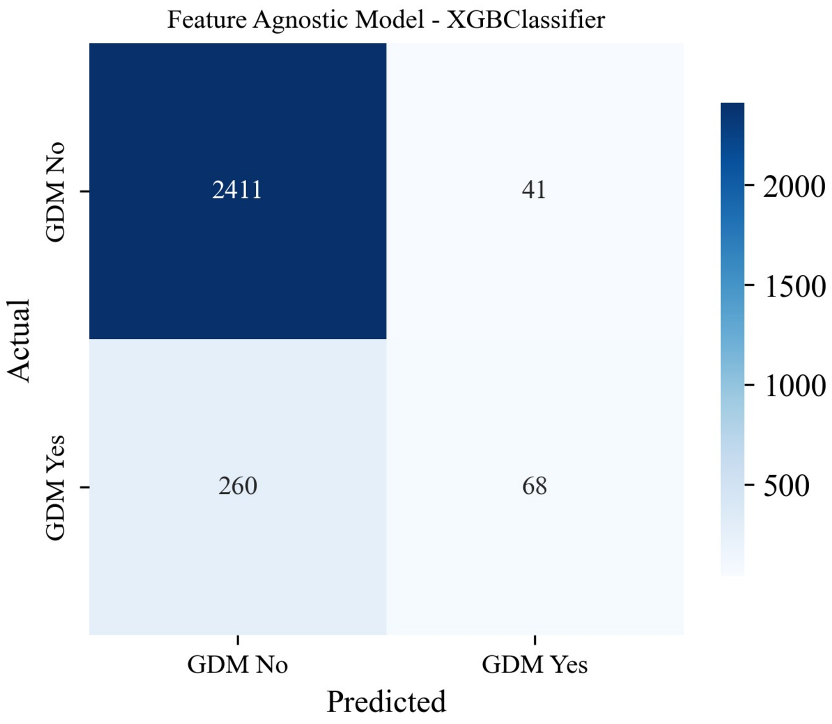
Feature Agnostic Model - XGBClassifier
Actual
GDM No
GDM Yes
2411
41
260
68
GDM No
GDM Yes
Predicted
2000
1500
1000
500

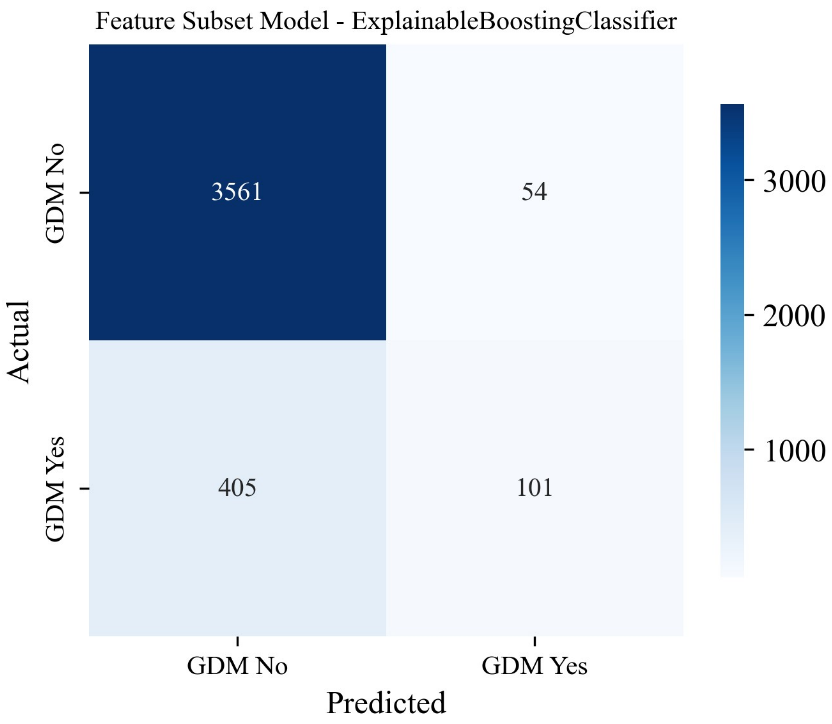
Feature Subset Model - ExplainableBoostingClassifier
Actual
GDM No
GDM Yes
3561
54
405
101
Predicted
GDM No
GDM Yes
3000
2000
1000

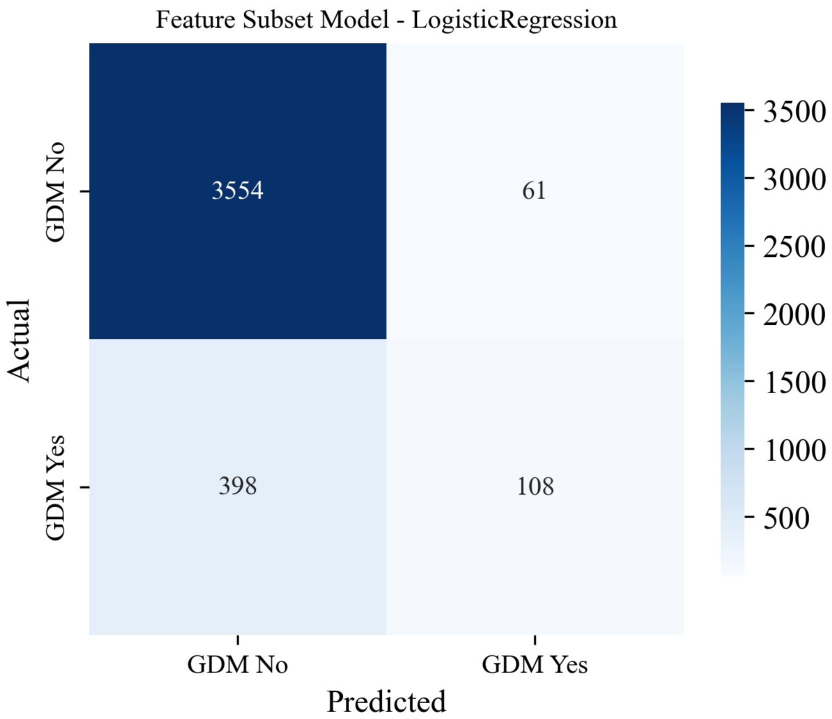
Feature Subset Model - LogisticRegression
Actual
GDM No
GDM Yes
3554
61
398
108
Predicted
GDM No
GDM Yes
3500
3000
2500
2000
1500
1000
500

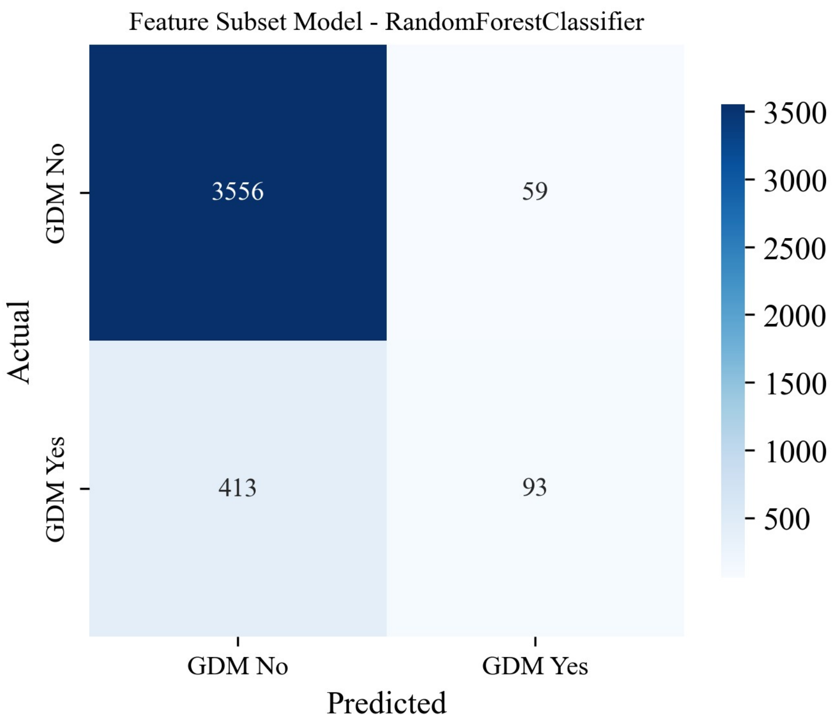
Feature Subset Model - RandomForestClassifier
Actual
GDM No
GDM Yes
3556
59
413
93
Predicted
GDM No
GDM Yes
3500
3000
2500
2000
1500
1000
500

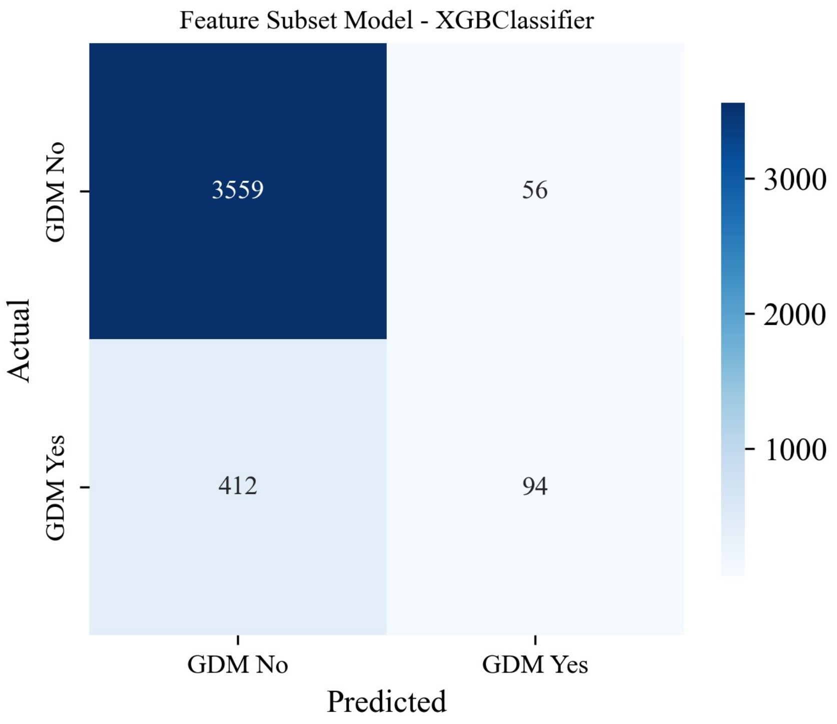
Feature Subset Model - XGBClassifier
Actual
GDM No
GDM Yes
3559
56
412
94
Predicted
GDM No
GDM Yes
3000
2000
1000

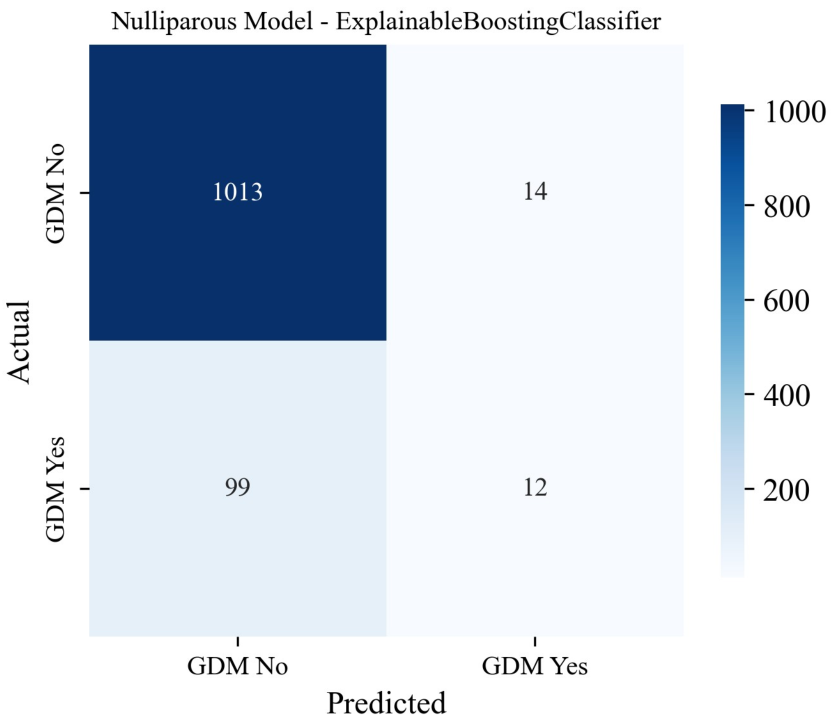
Nulliparous Model - ExplainableBoostingClassifier
Actual
GDM No
GDM Yes
1013
14
99
12
GDM No
GDM Yes
Predicted
1000
800
600
400
200

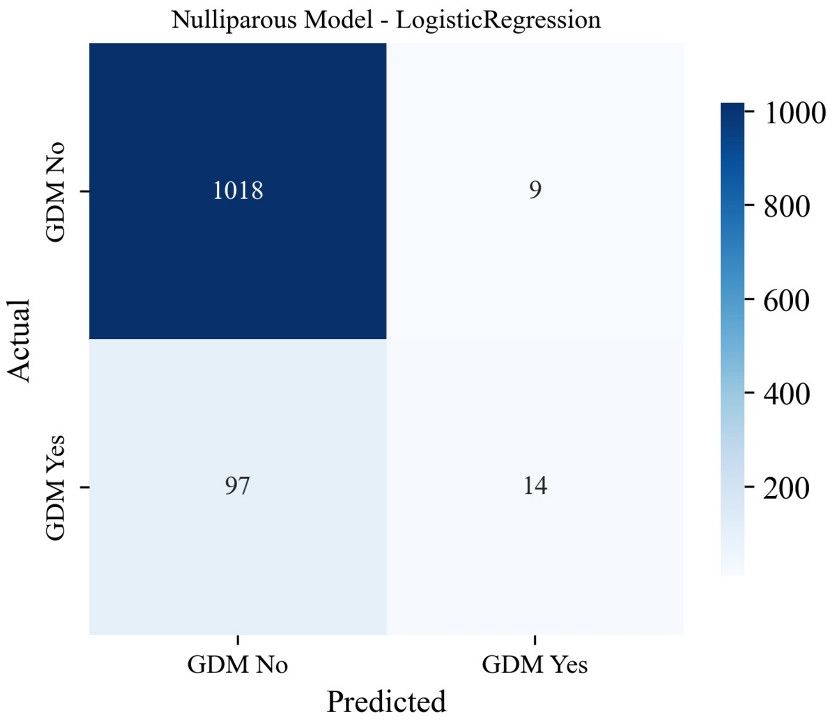
Nulliparous Model - LogisticRegression
Actual
GDM No
GDM Yes
1018
9
97
14
GDM No
GDM Yes
Predicted
1000
800
600
400
200

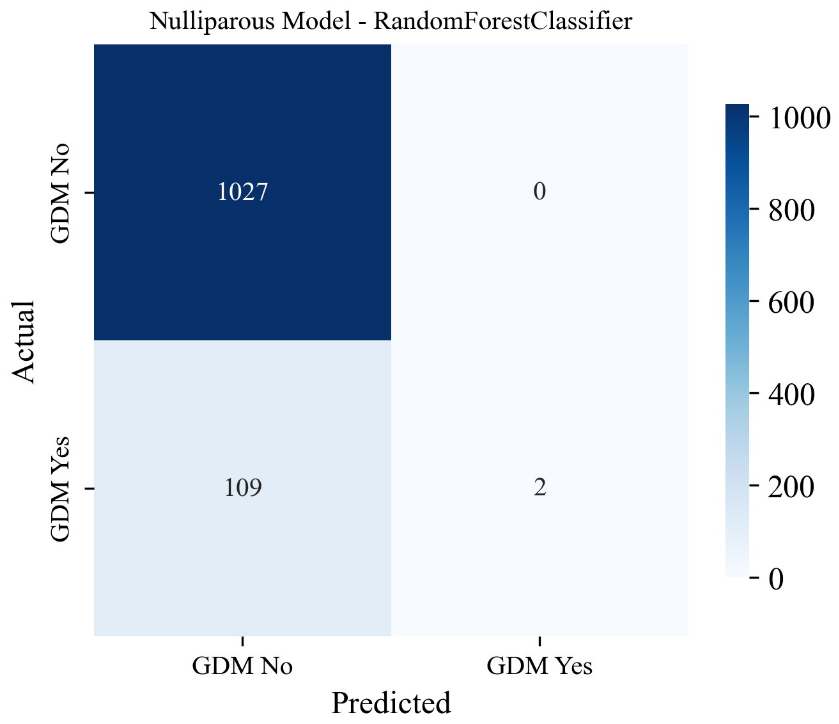
Nulliparous Model - RandomForestClassifier
Actual
GDM No
GDM Yes
1027
0
109
2
Predicted
GDM No
GDM Yes
1000
800
600
400
200
0

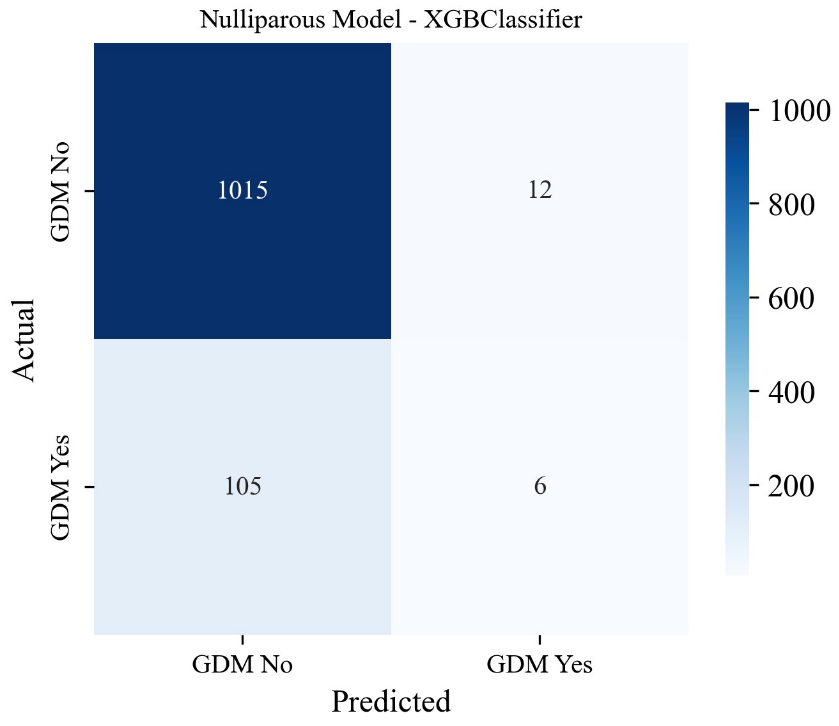
Nulliparous Model - XGBClassifier
Actual
GDM No
GDM Yes
1015
12
105
6
Predicted
GDM No
GDM Yes
1000
800
600
400
200

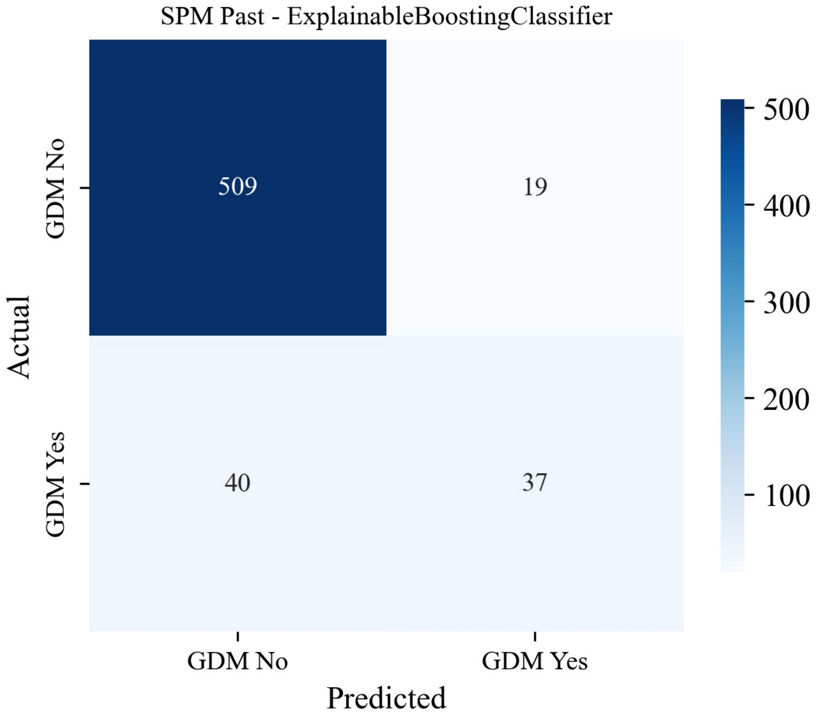
SPM Past - ExplainableBoostingClassifier
Actual
GDM No
GDM Yes
509
19
40
37
GDM No
GDM Yes
Predicted
500
400
300
200
100

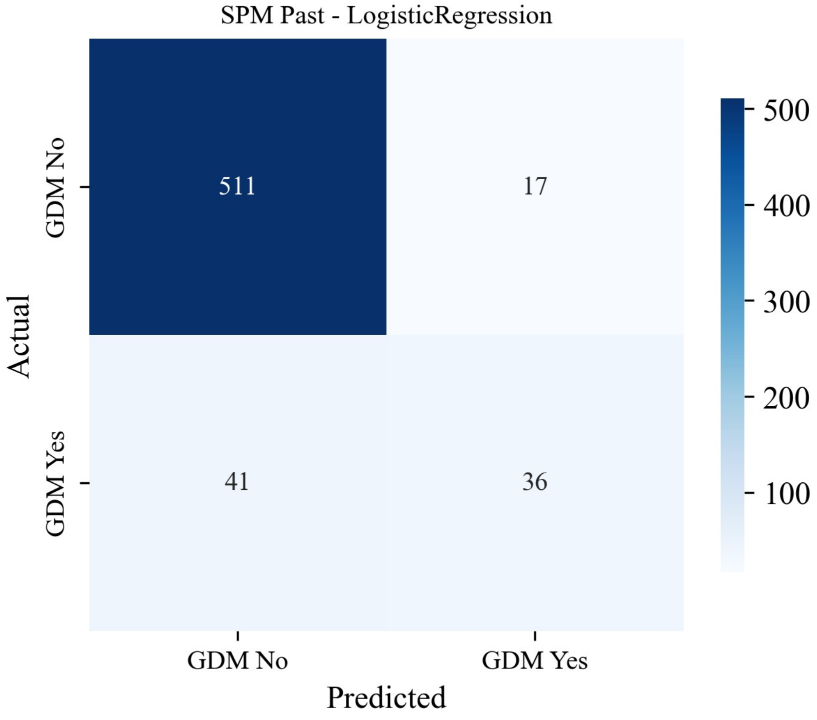
SPM Past - LogisticRegression
Actual
GDM No
GDM Yes
511
17
41
36
GDM No
GDM Yes
Predicted
500
400
300
200
100

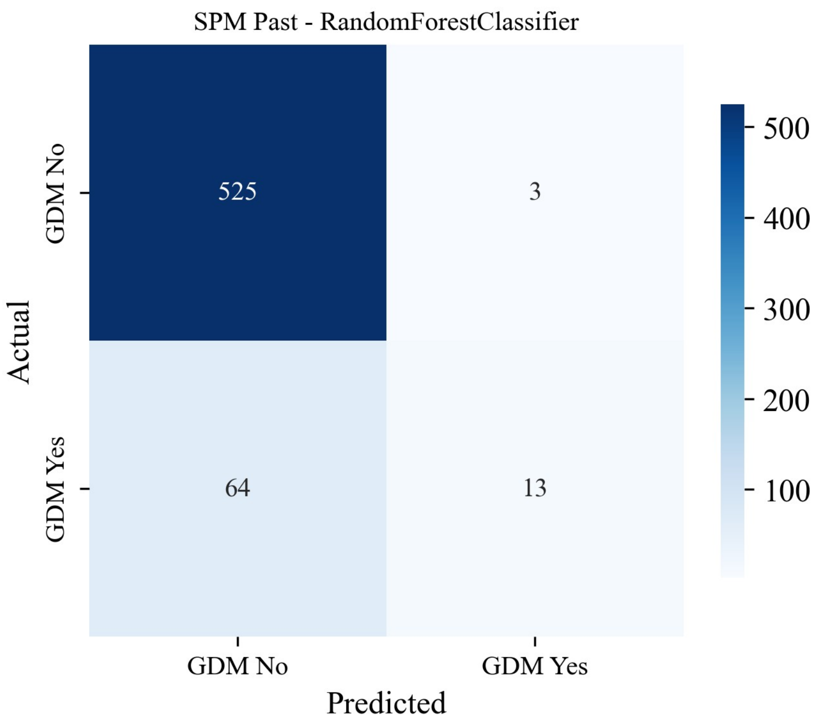
SPM Past - RandomForestClassifier
525
3
64
13
GDM No
GDM Yes
Actual
GDM No
GDM Yes
Predicted
500
400
300
200
100

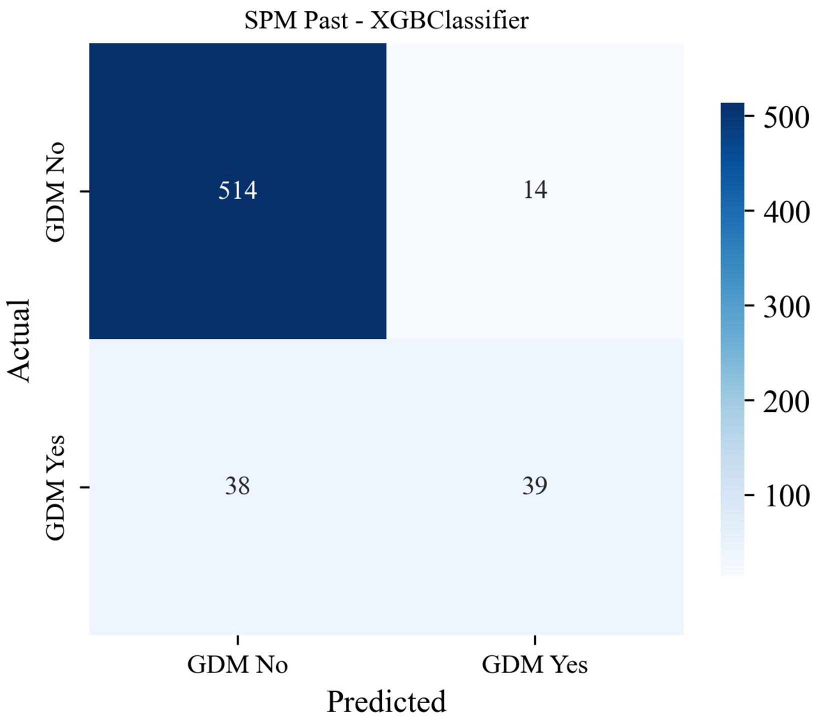
SPM Past - XGBClassifier
514
14
38
39
GDM No
GDM Yes
Actual
GDM No
GDM Yes
Predicted
500
400
300
200
100

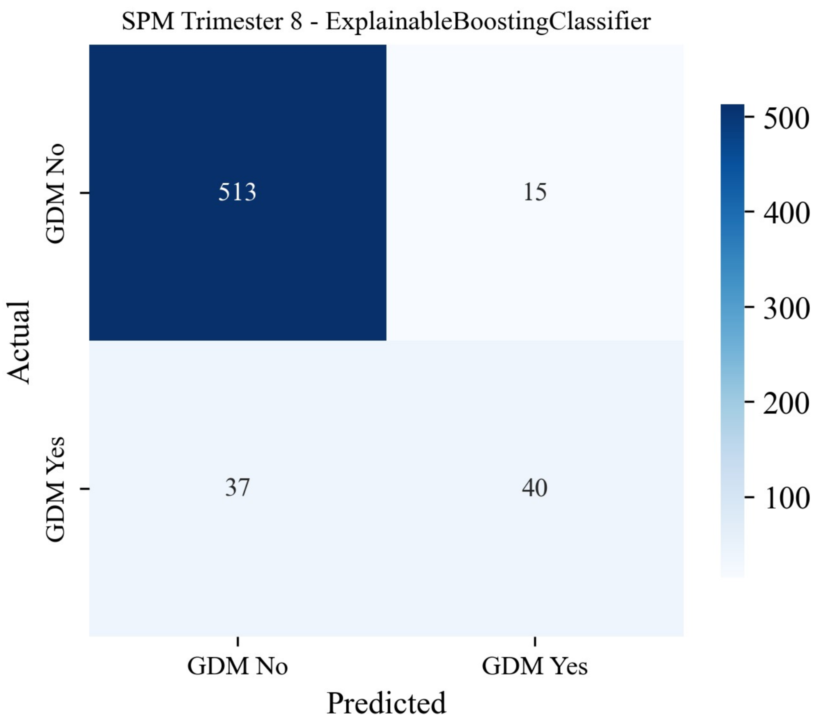
SPM Trimester 8 - ExplainableBoostingClassifier
Actual
GDM No
GDM Yes
513
15
37
40
Predicted
GDM No
GDM Yes
500
400
300
200
100

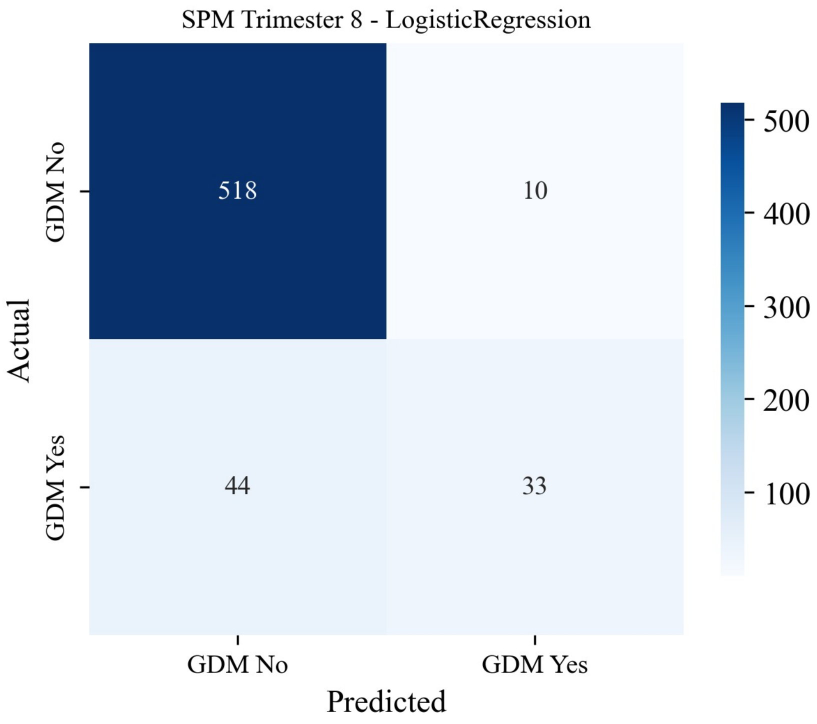
SPM Trimester 8 - LogisticRegression
Actual
GDM No
GDM Yes
518
10
44
33
Predicted
GDM No
GDM Yes
500
400
300
200
100

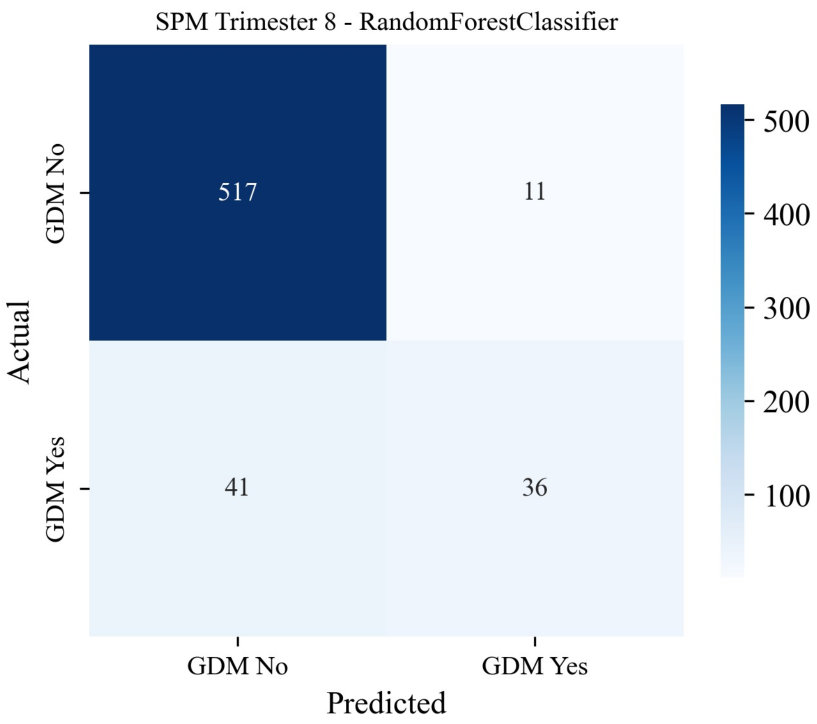
SPM Trimester 8 - RandomForestClassifier
Actual
GDM No
GDM Yes
517
11
41
36
Predicted
GDM No
GDM Yes
500
400
300
200
100

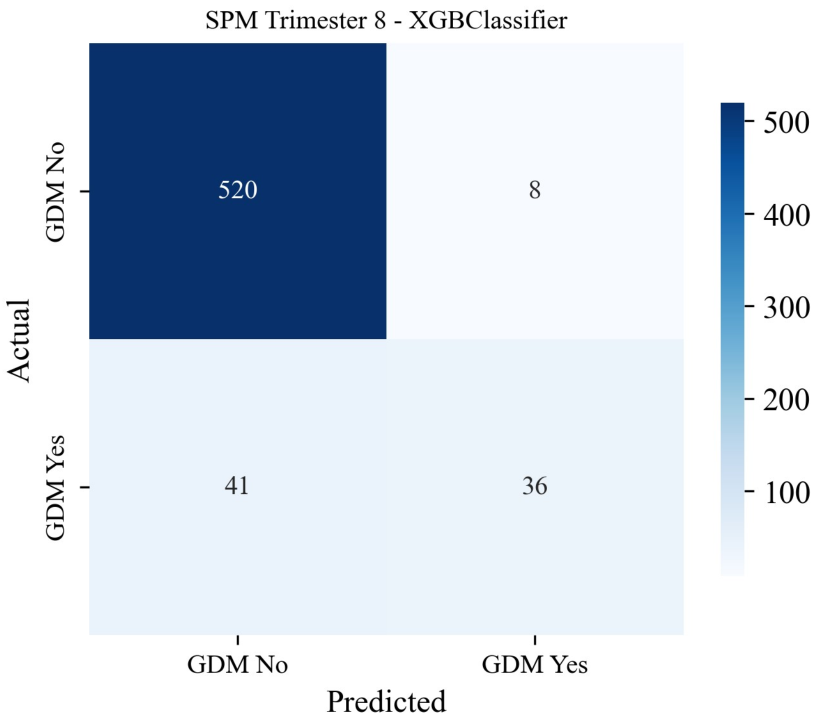
SPM Trimester 8 - XGBClassifier
Actual
GDM No
GDM Yes
520
8
41
36
Predicted
GDM No
GDM Yes
500
400
300
200
100

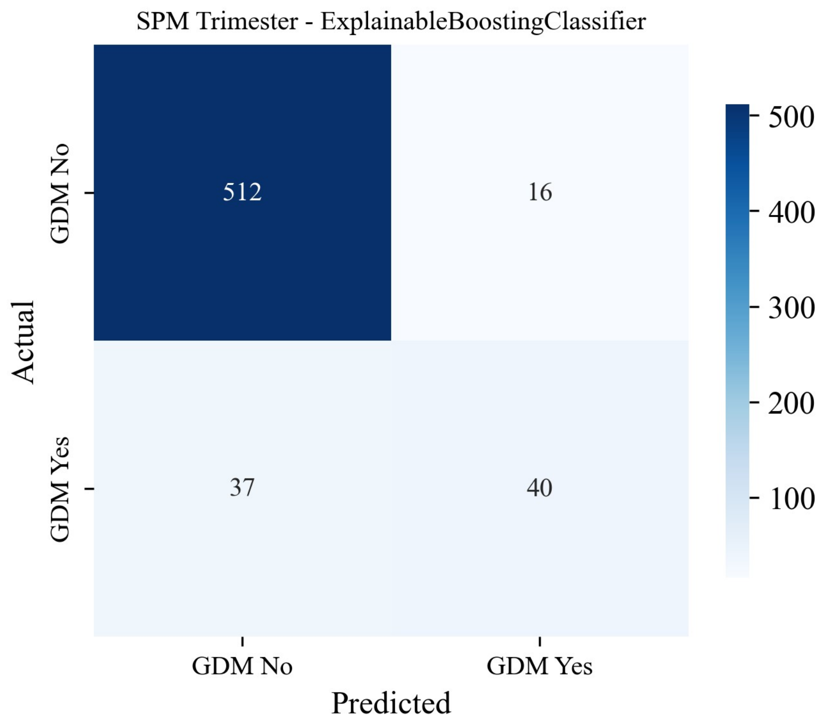
SPM Trimester - ExplainableBoostingClassifier
Actual
GDM No
GDM Yes
512
16
37
40
Predicted
GDM No
GDM Yes
500
400
300
200
100

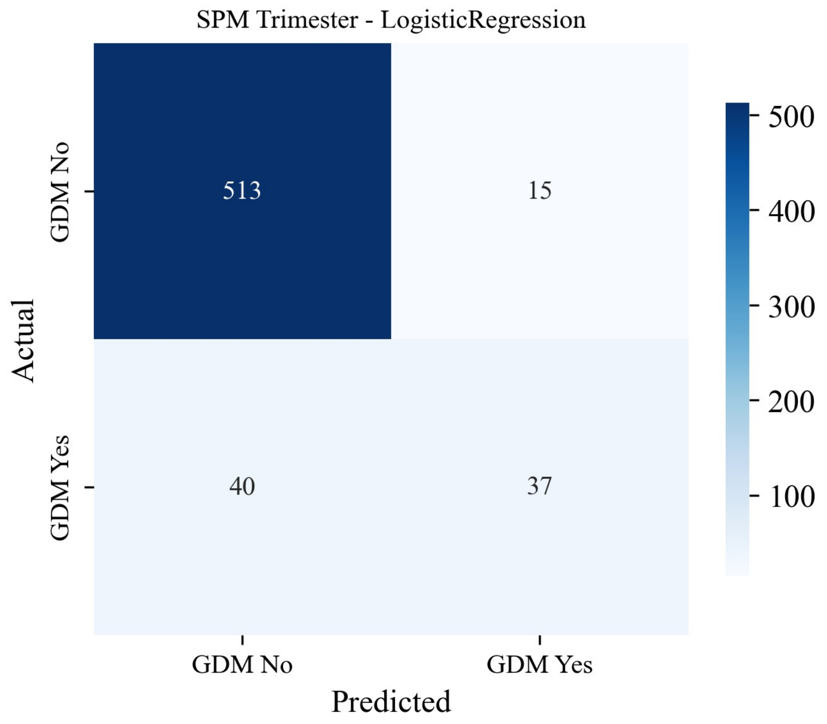
SPM Trimester - LogisticRegression
Actual
GDM No
GDM Yes
513
15
40
37
Predicted
GDM No
GDM Yes
500
400
300
200
100

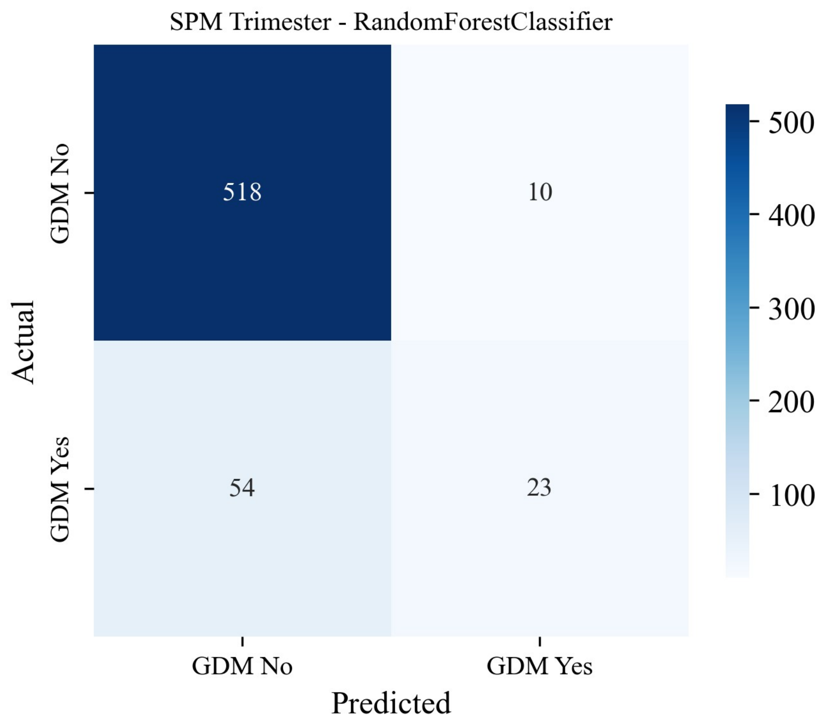
SPM Trimester - RandomForestClassifier
518
10
54
23
GDM No
GDM Yes
Actual
GDM No
GDM Yes
Predicted
500
400
300
200
100

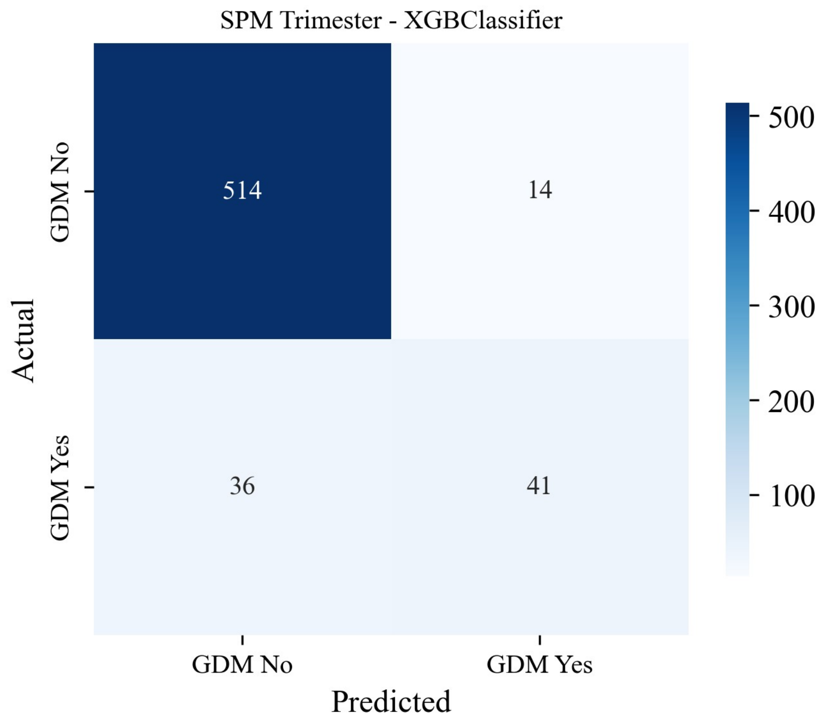
SPM Trimester - XGBClassifier
514
14
36
41
GDM No
GDM Yes
Actual
GDM No
GDM Yes
Predicted
500
400
300
200
100
