# Supplementary material for: Evaluation of machine learning models for early prediction of gestational diabetes using retrospective electronic health records from current and previous pregnancies
Source: BMJ Digit Health Ai. 2025 Dec 18;1(1):e000089. doi: 10.1136/bmjdhai-2025-000089 (PMC13492532; doi:10.1136/bmjdhai-2025-000089)
Supplement: Supplementary file 2 [file bmjdhai-1-1-bmjdhai-2025-000089-s002.docx]

**First Trimester XGBoost Model**

**First Trimester EBM Model**

**First Trimester RF Model**

**Nulliparous XGB Model**

**Nulliparous EBM Model**

**Nulliparous RF Model**

**Past Pregnancy XGB Model**

**Past Pregnancy EBM Model**

**Past Pregnancy RF Model**

**Multiparous XGB Model**

**Multiparous EBM Model**

**Multiparous RF Model**
