# Supplementary material for: Evaluation of machine learning models for early prediction of gestational diabetes using retrospective electronic health records from current and previous pregnancies
Source: BMJ Digit Health Ai. 2025 Dec 18;1(1):e000089. doi: 10.1136/bmjdhai-2025-000089 (PMC13492532; doi:10.1136/bmjdhai-2025-000089)
Supplement: Supplementary file 3 [file bmjdhai-1-1-bmjdhai-2025-000089-s003.docx]

**Supplementary Tables**

S Table 1. Hyperparameter space searched for the models during training

| Hyperparameter Search Space (n_iter = 10) | |
| --- | --- |
| **Random Forest Classifier** | - n_estimators: [100, 200, 300] - max_depth: [None, 10, 20, 30] - min_samples_split: [2, 5, 10] - min_samples_leaf: [1, 2, 4] |
| **Logistic Regression** | - C: np.logspace(-4, 4, 20) - solver: ['liblinear', 'lbfgs'] |
| **XGB Classifier** | - n_estimators: [100, 200, 300] - learning_rate: [0.01, 0.1, 0.2] - max_depth: [3, 4, 5, 10] |
| **Explainable Boosting Classifier** | - learning_rate: [0.01, 0.1, 1] - max_leaves: [3, 10, 20] |

S Table 2. Patient characteristics grouped by ethnicity expressed as number of occurrences (%).

| Characteristic | Caucasian n=24,180 | Southeast Asian  n=1,360 | Black African n=554 | Asian  n=489 | Middle Eastern n=154 | Other  n=824 |
| --- | --- | --- | --- | --- | --- | --- |
| **FH Diabetes^*^** |  |  |  |  |  |  |
| No | 19,161 (79.2) | 603 (44.3) | 390 (70.4) | 343 (70.1) | 83 (53.9) | 574 (69.7) |
| Yes | 5,019 (20.8) | 757 (55.7) | 164 (29.6) | 146 (29.9) | 71 (46.1) | 250 (30.3) |
| **Hx of GDM^*^** |  |  |  |  |  |  |
| No | 23,385 (96.7) | 1,187 (87.3) | 518 (93.5) | 465 (95.1) | 140 (90.1) | 788 (95.6) |
| Yes | 795 (3.3) | 173 (12.7) | 36 (6.5) | 24 (4.9) | 14 (9.9) | 36 (4.4) |
| **GDM** |  |  |  |  |  |  |
| No | 21,772 (90.0) | 899 (66.1) | 475 (85.7) | 383 (78.3) | 133 (86.4) | 711 (86.3) |
| Yes | 2,408 (10.0) | 461 (33.9) | 79 (14.3) | 106 (21.7) | 21 (13.6) | 113 (13.7) |
| **Parity^+^** |  |  |  |  |  |  |
| 0 | 10,022 (41.4) | 511 (37.6) | 229 (41.3) | 222 (45.4) | 54 (35.1) | 406 (49.3) |
| 1 | 8,741 (36.1) | 485 (35.7) | 167 (30.1) | 173 (35.4) | 51 (33.1) | 264 (32.0) |
| >=2 | 5,417 (22.4) | 364 (26.8) | 158 (28.5) | 94 (19.2) | 49 (31.8) | 154 (18.7) |
| **Age^#^** |  |  |  |  |  |  |
| Age (Mean ± SD) | 33 ± 5 | 32 ± 5 | 31 ± 6 | 33 ± 5 | 31 ± 5 | 33 ± 5 |
| >=40 | 1,934 (8.0) | 50 (3.7) | 50 (9.0) | 51 (10.4) | 4 (2.6) | 89 (10.8) |
| <40 | 22,246 (92.0) | 1,310 (96.3) | 504 (91.0) | 438 (89.6) | 150 (97.4) | 735 (89.2) |
| **BMI** |  |  |  |  |  |  |
| BMI (Mean ± SD) | 26.2 ± 4.3 | 26.1 ± 4.6 | 28.5 ± 6.1 | 24.2 ± 4.3 | 25.8 ± 4.0 | 25.6 ± 5.1 |
| <25 | 12,092 (50.0) | 601 (44.2) | 182 (32.9) | 316 (64.6) | 76 (49.4) | 439 (53.3) |
| 25 to <30 | 7,298 (30.2) | 540 (39.0) | 173 (31.2) | 117 (23.9) | 56 (37.0) | 237 (28.8) |
| 30 to <35 | 3,081 (12.7) | 176 (12.9) | 116 (20.9) | 44 (9.0) | 18 (11.7) | 102 (12.4) |
| 35 to <40 | 1,149 (4.8) | 36 (2.6) | 52 (9.4) | 10 (2.0) | 1 (0.6) | 32 (3.9) |
| >=40 | 560 (2.3) | 17 (1.3) | 31 (5.6) | 2 (0.4) | 2 (1.3) | 14 (1.7) |
| Systolic BP (Mean ± SD) | 111 ± 11 | 109 ± 10 | 113 ± 11 | 109 ± 11 | 107 ± 12 | 111 ± 10 |
| Diastolic BP (Mean ± SD) | 67 ± 8 | 67 ± 8 | 69 ± 9 | 66 ± 8 | 65 ± 8 | 67 ± 8 |

S Table 3. Performance of machine learning models predicting across the different ethnicity sub-groups. Performance measured by AUC evaluated using out-of-fold predictions. Minimum of 15 samples required.

| **Dataset and Model** | Caucasian | Southeast Asian | Black African | Asian | Middle Eastern | Other |
| --- | --- | --- | --- | --- | --- | --- |
| **First-Trimester Models** | n=24,180 | n=1,360 | n=554 | n=489 | n=154 | n=824 |
| Random Forest Classifier | 0.808 | 0.747 | 0.748 | 0.704 | 0.761 | 0.877 |
| Logistic Regression | 0.809 | 0.759 | 0.769 | 0.724 | 0.758 | 0.860 |
| XGBoost Classifier | 0.810 | 0.751 | 0.756 | 0.717 | 0.786 | 0.866 |
| Explainable Boosting Machine | 0.809 | 0.753 | 0.768 | 0.711 | 0.718 | 0.866 |
|  |  |  |  |  |  |  |
| **Nulliparous Models** | n=10,022 | n=511 | n=167 | n=222 | n=49 | n=406 |
| Random Forest Classifier | 0.804 | 0.727 | 0.700 | 0.747 | 0.803 | 0.865 |
| Logistic Regression | 0.811 | 0.727 | 0.722 | 0.731 | 0.854 | 0.870 |
| XGBoost Classifier | 0.810 | 0.732 | 0.697 | 0.719 | 0.752 | 0.855 |
| Explainable Boosting Machine | 0.805 | 0.729 | 0.668 | 0.741 | 0.786 | 0.864 |
|  |  |  |  |  |  |  |
| **Past Pregnancy Models** | n=3,648 | n=138 | n=70 | n=51 | n=19 | n=77 |
| Random Forest Classifier | 0.845 | 0.752 | 0.790 | 0.750 | 0.853 | 0.911 |
| Logistic Regression | 0.837 | 0.764 | 0.792 | 0.793 | 0.941 | 0.926 |
| XGBoost Classifier | 0.858 | 0.734 | 0.810 | 0.778 | 0.853 | 0.932 |
| Explainable Boosting Machine | 0.844 | 0.774 | 0.783 | 0.827 | 0.882 | 0.913 |
|  |  |  |  |  |  |  |
| **Multiparous Models** |  |  |  |  |  |  |
| Random Forest Classifier | 0.874 | 0.758 | 0.794 | 0.840 | 0.824 | 0.955 |
| Logistic Regression | 0.867 | 0.770 | 0.823 | 0.788 | 0.912 | 0.953 |
| XGBoost Classifier | 0.874 | 0.699 | 0.806 | 0.806 | 0.824 | 0.967 |
| Explainable Boosting Machine | 0.880 | 0.776 | 0.845 | 0.799 | 0.824 | 0.963 |

S Table 4. Final hyperparameters chosen for all models as a result of hyperparameter tuning. Only the tuned hyperparameters are displayed, all other parameters are thus default values.

| **Dataset and Model** | Hyperparameters | | | | | |
| --- | --- | --- | --- | --- | --- | --- |
| **First-Trimester Models** |  | | | | | |
| Random Forest Classifier | RandomForestClassifier(max_depth=10, min_samples_leaf=4) | | | | | |
| Logistic Regression | LogisticRegression(C=0.08858667904100823, max_iter=1000) | | | | | |
| XGBoost Classifier | XGBClassifier(max_depth=3, n_estimators=100, learning_rate=1) | | | | | |
| Explainable Boosting Machine | ExplainableBoostingClassifier() | | | | | |
|  |  |  |  |  |  |  |
| **FTP-9 Models** |  |  |  |  |  |  |
| Random Forest Classifier | RandomForestClassifier(max_depth=10, min_samples_leaf=4) | | | | | |
| Logistic Regression | LogisticRegression(C=0.23357214690901212, max_iter=1000, solver='liblinear') | | | | | |
| XGBoost Classifier | XGBClassifier(learning_rate=0.01, max_depth=5,n_estimators=300) | | | | | |
| Explainable Boosting Machine | ExplainableBoostingClassifier(learning_rate=0.1, max_bins=256) | | | | | |
|  |  |  |  |  |  |  |
| **Nulliparous Model** |  | | | | | |
| Random Forest Classifier | RandomForestClassifier(max_depth=10, min_samples_leaf=4) | | | | | |
| Logistic Regression | LogisticRegression(C=0.03359818286283781, max_iter=1000, solver='liblinear') | | | | | |
| XGBoost Classifier | XGBClassifier(max_depth=3, n_estimators=100, learning_rate=0.1) | | | | | |
| Explainable Boosting Machine | ExplainableBoostingClassifier(learning_rate=0.1, max_bins=64) | | | | | |
|  |  |  |  |  |  |  |
| **Sequential Model (Previous Pregnancy Variables)** |  | | | | | |
| Random Forest Classifier | RandomForestClassifier(max_depth=20, min_samples_leaf=4, min_samples_split=5,n_estimators=300) | | | | | |
| Logistic Regression | LogisticRegression(C=0.03359818286283781, max_iter=1000, solver='liblinear') | | | | | |
| XGBoost Classifier | XGBClassifier(max_depth=3, n_estimators=100, learning_rate=0.1) | | | | | |
| Explainable Boosting Machine | ExplainableBoostingClassifier(learning_rate=1) | | | | | |
|  |  |  |  |  |  |  |
| **Sequential Model (Including First Trimester Data)** |  |  |  |  |  |  |
| Random Forest Classifier | RandomForestClassifier(min_samples_leaf=2, n_estimators=300) | | | | | |
| Logistic Regression | LogisticRegression(C=0.03359818286283781, max_iter=1000, solver='liblinear') | | | | | |
| XGBoost Classifier | XGBClassifier(max_depth=10, n_estimators=100, learning_rate=0.1) | | | | | |
| Explainable Boosting Machine | ExplainableBoostingClassifier() | | | | | |
|  |  |  |  |  |  |  |
| **Sequential Model (Top 8 Features)** |  |  |  |  |  |  |
| Random Forest Classifier | RandomForestClassifier(max_depth=10, min_samples_split=5) | | | | | |
| Logistic Regression | LogisticRegression(C=0.08858667904100823, max_iter=1000) | | | | | |
| XGBoost Classifier | XGBClassifier(max_depth=5, n_estimators=300, learning_rate=0.01) | | | | | |
| Explainable Boosting Machine | ExplainableBoostingClassifier() | | | | | |

S Table 5. List, value count and ranges of all data available in the electronic health record during the first trimester.

| **Variable** | **Value Count/Range** |
| --- | --- |
| Ethnic Origin of Patient | {'CAUCASIAN': 24180, 'SOUTH EAST ASIAN': 1360, 'OTHER': 824, 'BLACK AFRICAN': 554, 'ASIAN': 489, 'MIDDLE EASTERN': 154} |
| Cardiac Problems | {'NO': 26027, 'YES': 1534} |
| Raised BP outside preg. | {'NO': 26959, 'IN PAST': 334, 'CURRENT': 268} |
| VV's/clotting probs. | {'NO': 25380, 'YES': 2181} |
| Urinary/kidney probs. | {'NO': 20874, 'YES': 6687} |
| Fits/epilepsy | {'NO': 23708, 'YES': 3853} |
| Jaundice/liver disease | {'TATTOO/PIERCING': 14834, 'NO': 12211, 'YES': 516} |
| Resp. system disease | {'NO': 22588, 'YES': 4973} |
| Metabolic Disorders | {'NO': 27537, 'YES': 24} |
| Digestive tract disease | {'NO': 23209, 'YES': 4352} |
| Serious infections | {'NO': 20883, 'YES': 6678} |
| Connective Tissue | {'NO': 27167, 'YES': 394} |
| Orthopaedic Probs | {'NO': 20212, 'YES': 7349} |
| Operations in past | {'NO': 18217, 'YES': 9344} |
| Gynae. probs./operations | {'NO': 16207, 'YES': 11354} |
| STD - ever | {'NO': 20295, 'YES': 7266} |
| Anaemia/blood disorder | {'NO': 20464, 'YES': 7097} |
| Anaemia Treatment | {'NONE': 21447, 'YES': 6114} |
| Blood transfusion - ever | {'NO': 26354, 'YES': 1207} |
| Blood/Blood products | {'YES': 27504, 'NO': 57} |
| Cigarette Alternatives | {'NO': 26611, 'YES': 950} |
| Folic acid | {'POSTCONCEPTION': 12503, 'BOTH': 10791, 'PRECONCEPTION': 3439, 'NO': 828} |
| Folic acid length | {'0-3 months': 14520, '3-6 months': 6852, '6-12 months': 3165, 'longer than 12 months': 3024} |
| Psychological prob | {'NO': 20623, 'YES': 6938} |
| Illicit drugs ever | {'NO': 25487, 'YES': 2074} |
| Illicit Drugs - Partner | {'NO': 26458, 'YES': 1103} |
| Allergies | {'NO': 21558, 'YES': 6003} |
| FH Hypertension | {'NO': 14316, 'YES': 13245} |
| FH of Cancer | {'NO': 19806, 'YES': 7755} |
| FH TB | {'NO': 27241, 'YES': 320} |
| FH Blood disorder | {'NO': 24516, 'YES': 3045} |
| FH - Heart Problems | {'NO': 16342, 'YES': 11219} |
| FH Thyroidism | {'NO': 21533, 'YES': 6028} |
| FH Diabetes | {'NO': 21154, 'YES': 6407} |
| FH Multiple preg. | {'NO': 20717, 'YES': 6844} |
| FH Mental Illness | {'NO': 23890, 'YES': 3671} |
| FH-Congenital Abnormality | {'NO': 21730, 'YES': 5831} |
| Pregnant Before | {'YES': 18867, 'NO': 8694} |
| Misc/TOP/Ect Before | {'NO': 18333, 'YES': 9228} |
| Pregnant no of Times | {'min': 0, 'max': 16, 'range': 16} |
| Proteinuria at booking | {'NO': 27433, 'YES': 128} |
| Systolic BP at booking | {'min': 58, 'max': 195, 'range': 137} |
| Diastolic BP at booking | {'min': 40.0, 'max': 120.0, 'range': 80.0} |
| Planned pregnancy | {'PLANNED PREGNANCY': 19027, 'UNPLANNED PREGNANCY': 6719, 'INFERTILITY TREATMENT': 1815} |
| Infertility Treatment | {'NO': 25791, 'YES': 1770} |
| Pregnant on contraception | {'NO': 26344, 'YES': 1217} |
| Cycle Regular | {'YES': 21789, 'NO': 5772} |
| Tests/investigations | {'NO': 15312, 'YES - OTHER': 9593, 'EPAU': 2656} |
| Weight at Booking | {'min': 32.4, 'max': 175.2, 'range': 142.79999999999998} |
| Gravida | {'min': 0.0, 'max': 16.0, 'range': 16.0} |
| Parity (not inc.multiple) | {'min': 0, 'max': 11, 'range': 11} |
| Livebirths - No | {'min': 0.0, 'max': 11.0, 'range': 11.0} |
| Stillbirths - No | {'min': 0, 'max': 2, 'range': 2} |
| Neonatal deaths - No | {'min': 0, 'max': 2, 'range': 2} |
| Deaths after 28 days - No | {'min': 0, 'max': 2, 'range': 2} |
| Caesarean Sections - No | {'min': 0, 'max': 6, 'range': 6} |
| Terminations - No | {'min': 0, 'max': 4, 'range': 4} |
| Miscarriages - No | {'min': 0, 'max': 11, 'range': 11} |
| Ectopics - No | {'min': 0, 'max': 3, 'range': 3} |
| Previous moles - No. | {'min': 0, 'max': 1, 'range': 1} |
| Drug Abuse | {'NO': 27465, 'YES': 96} |
| Fertility treatment | {'NO': 25815, 'YES': 1746} |
| Hb Electrophoresis | {'NOT PERFORMED': 25535, 'NORMAL': 1578, 'ABNORMAL': 448} |
| Serology Result | {'NEGATIVE': 27319, 'NOT PERFORMED': 152, 'POSITIVE': 90} |
| H.I.V. Status | {'NEGATIVE': 27463, 'UNKNOWN': 54, 'POSITIVE': 44} |
| Last Haemoglobin | {'min': 6.1, 'max': 18.7, 'range': 12.6} |
| Bleeding during Pregnancy | {'NO': 24292, 'YES': 3269} |
| Hypertension during preg | {'NO': 26176, 'YES': 1385} |
| Hypertension Treatment | {'NONE': 26603, 'YES': 958} |
| Fetal problems | {'NO': 22080, 'YES OTHER': 4651, 'MACROSOMIA': 830} |
| Scan Abnormalities | {'NO': 26936, 'YES OTHER': 414, 'LGA': 127, 'SGA': 84} |
| Age at booking | {'min': 15, 'max': 53, 'range': 38} |
| GDM | {'min': 0, 'max': 1, 'range': 1} |
| Height of Mother (m) | {'min': 1.26, 'max': 1.95, 'range': 0.69} |
| BMI | {'min': 14.5, 'max': 61.1, 'range': 46.6} |
| Smoke Now | {'NO': 16465, 'STOPPED': 8636, 'YES': 2460} |
| Ever Smoked | {'NO': 16459, 'YES': 11102} |
| Skill Level | {'min': 0, 'max': 4, 'range': 4} |
| Hx_GDM | {'min': 0, 'max': 1, 'range': 1} |
| Other Endocrine probs | {'min': 0, 'max': 1, 'range': 1} |

S Table 6. Comparative performance metrics of machine learning models trained using the full feature set and evaluated via out-of-fold predictions across multiple datasets. AUC (Area Under the Receiver Operating Characteristic Curve), AP (Average Precision Score), O:E Ratio (Observed to Expected Ratio).

| **Dataset and Model** | **AUC (95% CI)** | **Calibration Slope** | **Calibration Intercept** | **O:E Ratio** | | **AP** | **Sensitivity** | **Specificity** | **F1 Score** | **Brier Score** |
| --- | --- | --- | --- | --- | --- | --- | --- | --- | --- | --- |
| **First-Trimester Models** |  |  |  |  |  | |  |  |  |  |
| Random Forest Classifier | 0.819 (0.810–0.827) | 1.194 | 0.288 | 0.986 | 0.440 | | 0.141 | 0.993 | 0.236 | 0.083 |
| Logistic Regression | 0.823 (0.815–0.831) | 0.998 | -0.004 | 0.999 | 0.443 | | 0.218 | 0.983 | 0.324 | 0.082 |
| XGBoost Classifier | 0.824 (0.816–0.832) | 1.034 | 0.057 | 1.001 | 0.442 | | 0.192 | 0.987 | 0.298 | 0.082 |
| Explainable Boosting Machine | 0.822 (0.814–0.830) | 0.961 | -0.055 | 1.006 | 0.443 | | 0.212 | 0.985 | 0.320 | 0.082 |
| Dummy Classifier | 0.508 (0.489–0.527) | 0.002 | -1.953 |  | 0.120 | | 0.128 | 0.888 | 0.130 | 0.201 |
|  |  |  |  |  |  | |  |  |  |  |
| **Nulliparous Models** |  |  |  |  |  | |  |  |  |  |
| Random Forest Classifier | 0.810 (0.796–0.824) | 1.311 | 0.568 | 1.000 | 0.326 | | 0.005 | 0.999 | 0.011 | 0.077 |
| Logistic Regression | 0.818 (0.804–0.831) | 1.036 | 0.052 | 0.989 | 0.347 | | 0.105 | 0.990 | 0.176 | 0.076 |
| XGBoost Classifier | 0.816 (0.803–0.829) | 1.004 | -0.018 | 1.009 | 0.337 | | 0.066 | 0.993 | 0.117 | 0.076 |
| Explainable Boosting Machine | 0.812 (0.799–0.825) | 0.878 | -0.134 | 1.067 | 0.343 | | 0.135 | 0.987 | 0.215 | 0.076 |
| Dummy Classifier | 0.503 (0.474–0.535) | 0.001 | -2.196 |  | 0.098 | | 0.108 | 0.899 | 0.106 | 0.178 |
|  |  |  |  |  |  | |  |  |  |  |
| **Past Pregnancy Models** |  |  |  |  |  | |  |  |  |  |
| Random Forest Classifier | 0.854 (0.833–0.872) | 1.480 | 0.679 | 0.980 | 0.556 | | 0.163 | 0.993 | 0.268 | 0.077 |
| Logistic Regression | 0.848 (0.826–0.866) | 1.018 | -0.011 | 0.976 | 0.548 | | 0.425 | 0.969 | 0.514 | 0.073 |
| XGBoost Classifier | 0.861 (0.841–0.879) | 1.053 | 0.059 | 0.986 | 0.591 | | 0.423 | 0.976 | 0.530 | 0.071 |
| Explainable Boosting Machine | 0.855 (0.835–0.873) | 0.961 | -0.030 | 1.019 | 0.570 | | 0.445 | 0.971 | 0.570 | 0.072 |
| Dummy Classifier | 0.459 (0.429–0.492) | -0.013 | -2.290 |  | 0.123 | | 0.065 | 0.852 | 0.062 | 0.248 |
|  |  |  |  |  |  | |  |  |  |  |
| **Multiparous Models** |  |  |  |  |  | |  |  |  |  |
| Random Forest Classifier | 0.875 (0.858–0.891) | 1.445 | 0.547 | 0.962 | 0.600 | | 0.229 | 0.989 | 0.351 | 0.073 |
| Logistic Regression | 0.868 (0.849–0.884) | 0.980 | -0.051 | 0.988 | 0.558 | | 0.437 | 0.971 | 0.530 | 0.072 |
| XGBoost Classifier | 0.887 (0.870–0.902) | 0.606 | -0.050 | 1.318 | 0.637 | | 0.460 | 0.976 | 0.637 | 0.070 |
| Explainable Boosting Machine | 0.890 (0.873–0.905) | 0.824 | -0.113 | 1.074 | 0.638 | | 0.487 | 0.976 | 0.586 | 0.066 |
| Dummy Classifier | 0.459 (0.429–0.492) | -0.013 | -2.290 |  | 0.123 | | 0.065 | 0.852 | 0.062 | 0.248 |
